# Molecular and physiological responses to desiccation indicate the abscisic acid pathway is conserved in the peat moss, *Sphagnum*

**DOI:** 10.1093/jxb/erac133

**Published:** 2022-04-06

**Authors:** Candida Nibau, Willem van de Koot, Dominic Spiliotis, Kevin Williams, Tina Kramaric, Manfred Beckmann, Luis Mur, Yuji Hiwatashi, John H Doonan

**Affiliations:** National Plant Phenomics Centre, Institute of Biological, Environmental and Rural Sciences, Aberystwyth University, Aberystwyth, UK; National Plant Phenomics Centre, Institute of Biological, Environmental and Rural Sciences, Aberystwyth University, Aberystwyth, UK; National Plant Phenomics Centre, Institute of Biological, Environmental and Rural Sciences, Aberystwyth University, Aberystwyth, UK; National Plant Phenomics Centre, Institute of Biological, Environmental and Rural Sciences, Aberystwyth University, Aberystwyth, UK; Institute of Biological, Environmental and Rural Sciences, Aberystwyth University, Aberystwyth, UK; Institute of Biological, Environmental and Rural Sciences, Aberystwyth University, Aberystwyth, UK; Institute of Biological, Environmental and Rural Sciences, Aberystwyth University, Aberystwyth, UK; School of Food Industrial Sciences, Miyagi University, Sendai, Japan; National Plant Phenomics Centre, Institute of Biological, Environmental and Rural Sciences, Aberystwyth University, Aberystwyth, UK; University of Exeter, UK

**Keywords:** ABA, chlorophyll fluorescence, desiccation, drought, moss, peat, relative water content, *Sphagnum*

## Abstract

Mosses of the genus *Sphagnum* are the main components of peatlands, a major carbon-storing ecosystem. Changes in precipitation patterns are predicted to affect water relations in this ecosystem, but the effect of desiccation on the physiological and molecular processes in *Sphagnum* is still largely unexplored. Here we show that different *Sphagnum* species have differential physiological and molecular responses to desiccation but, surprisingly, this is not directly correlated with their position in relation to the water table. In addition, the expression of drought responsive genes is increased upon water withdrawal in all species. This increase in gene expression is accompanied by an increase in abscisic acid (ABA), supporting a role for ABA during desiccation responses in *Sphagnum*. Not only do ABA levels increase upon desiccation, but *Sphagnum* plants pre-treated with ABA display increased tolerance to desiccation, suggesting that ABA levels play a functional role in the response. In addition, many of the ABA signalling components are present in *Sphagnum* and we demonstrate, by complementation in *Physcomitrium patens*, that *Sphagnum* ABI3 is functionally conserved. The data presented here, therefore, support a conserved role for ABA in desiccation responses in *Sphagnum*.

## Teaser

Abscisic acid is a key conserved physiological component in the molecular mechanisms underlying desiccation responses in the moss *Sphagnum*.

## Introduction

One of the biggest challenges facing primordial plants as they moved to terrestrial environments was how to cope with water stress. Arguably, the most important development of the water-to-land transition was the development of systems that allowed the plant to maintain an internal water potential independently from the water potential of the environment. These include the development of vascular systems, physical barriers to water loss in the form of cuticles, and complex drought-responsive cellular signalling pathways, all features of vascular plants. However, some plants developed strategies to avoid water stress by living in water-saturated terrestrial environments, increasing plant density or reducing plant size to reduce evaporation. Others evolved physiological mechanisms to tolerate short periods of drought and a few can survive nearly full desiccation. Despite this, a degree of tolerance to water loss is necessary in all the habitats that are not constantly water saturated. Even short periods of mild drought have deleterious effects in terms of plant growth and development, and plants need to develop strategies to mitigate these. Understanding these strategies is even more important given climate change scenarios in which temperature and rainfall are predicted to become more stochastic.

Traditionally, plant responses to drought have been defined as escape (accelerating reproduction), avoidance (developing strategies to maintain high internal water content), and tolerance (maintaining growth under low internal water content). Drought tolerance has been extensively studied in vascular plants and involves the activation of mechanisms to prevent water loss and increase water uptake, and pathways leading to the induction of drought responsive genes (Chaves *et al.*, 2003; Yao *et al.*, 2021). Central to these tolerance processes is the stress hormone abscisic acid (ABA). ABA rapidly increases at the onset of drought and triggers a complex and multi-layered signalling cascade leading to the transcription of drought responsive genes that enable the plant to maintain and adapt physiological processes under drought (Ali *et al.*, 2020).

Although most attention has focused on the effect of drought on agricultural systems, drought also poses a significant threat to wetland environments such as peatlands (Stirling *et al.*, 2020). Peatlands are important carbon-storing ecosystems in temperate and boreal regions and they are now seen as crucial to climate change mitigation strategies (Leifeld and Menichetti, 2018). Reversing human impact on peatland degradation often involves restoring the water table and vegetational cover on bare peat surfaces. However, response of key plants in these peatland ecosystems to drought is poorly understood.

Mosses of the genus *Sphagnum* are major components of acidic peatlands where they regulate water relations and carbon sequestration through peat accumulation. In common with all bryophytes, *Sphagnum* mosses are poikilohydric meaning that they are unable to maintain internal water potential different from their environment (Raven, 1995). Although typical mosses lack many of the adaptations of vascular plants to reduce water loss such as cuticle and stomata, they have developed alternative specialized features to store and regulate water distribution across the plant (Proctor *et al.*, 2007; Charron and Quatrano, 2009). These include morphological adaptations such as long and densely packed branches, growing in tightly packed cushions to prevent evaporation, and the development of specialized water storage cells that use water tension to keep the plant hydrated while minimally affecting gas exchange (Dilks and Proctor, 1979; Proctor, 2000; Proctor *et al.*, 2007). Physiological adaptations include the development of desiccation tolerance that enables mosses to recover after completely drying out (Oliver *et al.*, 2005). This is achieved through the presence of constitutive mechanisms of cellular protection and a rehydration-induced repair and recovery process (Proctor *et al.*, 2007). These mechanisms are well studied in the moss *Tortula ruralis*, where the constitutive expression of Late Embryogenic Abundant (LEA) proteins is essential for cellular protection and maintaining membrane integrity during drying (Oliver *et al.*, 2005). The development of desiccation avoidance and tolerance mechanisms may have allowed mosses to colonize a wide range of habitats across all continents, making the group very successful and resilient (Charron and Quatrano, 2009).

Unlike most bryophytes, *Sphagnum* species have been considered to be drought and desiccation-intolerant (Clymo, 1973; Clymo and Hayward, 1982). More recently, this view has been challenged, and while some authors report low recovery even after mild desiccation, other studies suggest that *Sphagnum* shoots can tolerate strong desiccation (reviewed in Hájek and Vicherová, 2014). The ability (or otherwise) of *Sphagnum* to survive water-limiting conditions would have implications for the restoration and management of peatland ecosystems. With predicted changes in climatic patterns, peatlands are considered to be key to mitigation strategies. Therefore, understanding how *Sphagnum* mosses respond to drought and desiccation is necessary and timely (Leifeld and Menichetti, 2018).

There may be intrinsic variation in desiccation tolerance within the genus. A typical peatland consists of a variety of microhabitats ranging from permanently flooded pools, wet depressions (hollows), elevated hummocks, and everything in between. Access to the water table in these microhabitats is different and it has been proposed that the *Sphagnum* species that inhabit each habitat have evolved different mechanisms to cope with changes in water availability (Johnson *et al.*, 2015). Thus, hummock species that grow further away from the water table are expected to be better at maintaining colony water content and thus avoiding desiccation, while hollow species with constant access to the water table would be less desiccation tolerant. Experimental evidence seems to show that this is not always the case, with some studies finding no differences between position along the water table and desiccation tolerance (Wagner and Titus, 1984) while others find that hollow species actually show better recovery after desiccation (Schipperges and Rydin, 1998).

While some of the physiological mechanisms for *Sphagnum* response to water limiting conditions have been investigated, including the loss and recovery of photosynthetic function, not much is known about the molecular responses to desiccation (Wagner and Titus, 1984; Schipperges and Rydin, 1998; Hájek and Vicherová, 2014; Winnicka and Melosik, 2019). It has previously been shown that drought responsive gene expression is up-regulated in *Sphagnum* when subjected to drought and that ABA increases desiccation tolerance in *Sphagnum* (Marschall and Borbely, 2011; Hájek and Vicherová, 2014; Winnicka and Melosik, 2019). Despite this, a systematic and comparative analysis including both physiological and molecular responses of different *Sphagnum* species from different microhabitats to controlled drought and rehydration is lacking.

Here we show that desiccation responses differ in different *Sphagnum* species from the same blanket bog. Photosynthetic function and water loss are different between the species and this translates into differential activation of drought responsive gene expression. Our data confirm that ABA pre-treatment improves desiccation tolerance in *Sphagnum* and, significantly, that ABA levels increase upon desiccation in *Sphagnum.* Finally, we show that components of the ABA signalling pathway in *Sphagnum* are functional when expressed in *Physcomitrium* (*Physcomitrella*) *patens*. Our results therefore support a functional role for ABA and ABA responsive genes in desiccation responses in *Sphagnum*.

## Materials and methods

### Site collection and plant material

**Fig. 1.**
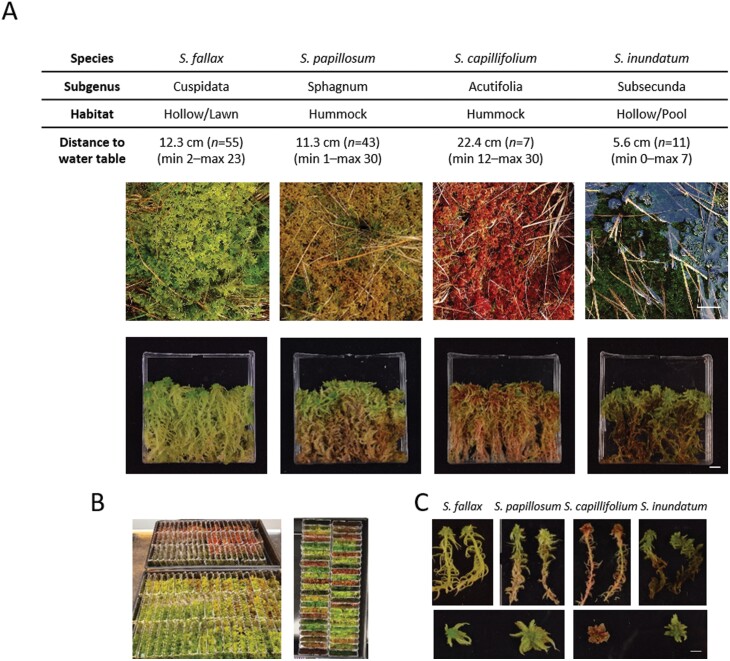
Details of the species used in this study and experimental set-up. (A) Four *Sphagnum* species were used, *S. fallax*, *S. papillosum*, *S. capillifolium*, and *S. inundatum*, belonging to the most abundant subgenera and spanning a range of microhabitats as indicated. Mean distances to the water table, as measured over 3 years at the Pen y Garn site, are also shown. Top images show the species in the field (scale bar: 10 cm) and bottom images after they have been assembled into square Petri dishes opened at the top to allow evaporation (cosms) at a density comparable to that observed in the field (scale bar: 1 cm). (B) Desiccation treatment set-up. The cosms were placed upright in a tray and kept in the greenhouse under natural light conditions. (C) Morphology of the different *Sphagnum* species as indicated. Top row, whole plants; bottom row detached capitula detail (scale bar: 1 cm).

*Sphagnum* species were collected from a minerotrophic mire in Pen y Garn (SN791758) situated in the Cambrian Mountains, Wales, UK. The mire is located at the base of a mountain and contains dense carpets of *Sphagnum* as well as several pool areas that exhibit mild flow after heavy rain. Four *Sphagnum* species, *Sphagnum fallax* (Cuspidata), *Sphagnum papillosum* (Sphagnum), *Sphagnum capillifolium* (Acutifolia), and *Sphagnum inundatum* (Subsecunda), were selected to cover the four most abundant taxonomic sections within the genus and the different habitats within the mire (Fig. 1). For each species, four different areas within the bog were selected for collection. In each area, stem density was manually scored and the height of the water table measured. Blocks of moss were lifted, carefully retaining the structural coherence of the canopy, and brought back to the laboratory. Data shown here are from a collection on 3 May 2021 but we collected at different times during the 2020–2021 season with similar results.

### Desiccation experiment

The desiccation experiments were performed in plastic square Petri dishes (cosms, Fig. 1) where one side of both the lid and the base were removed so that there is a 20 cm^2^ opening. The plates were positioned vertically so that the opening was uppermost. The same day they were collected, *Sphagnum* stems from the blocks were separated, cut to a length of 7 cm, and placed in each cosm at the same density as determined in the field. Each *S. fallax* cosm had 33 stems, *S. papillosum* 24, *S. capillifolium* 38, and *S. inundatum* 12. We prepared cosms for eight independent time points and four replicate cosms per time point in a total of 64 cosms per species. Each cosm was weighed to make sure they were all approximately the same weight. The cosms were then placed in a tub filled with water so that about two-thirds of the cosm was submerged and left overnight, allowing for full hydration of the moss. The following morning the excess water was allowed to drain from the plates for 1 h. All the cosms were weighed (starting weight) and chlorophyll fluorescence measured (see below). Four replicates (0 d) were taken and capitula harvested for relative water content (RWC) measurements, ABA analysis and RNA extraction. Plates were again weighed, imaged, and four replicates collected after 2, 4, 7, and 9 d for *S. fallax*, *S. capillifolium*, and *S. inundatum*. After 9 d water withdrawal, four cosms for these three species were placed in a tub of water and allowed to re-hydrate for 24 h and then the excess water allowed to drain for 1 h, after which plates were weighed, imaged, and harvested as before (re-wet time point). For *S. papillosum* samples were collected at 2, 4, 7, 9, and 13 d after water withdrawal before re-watering.

### Chlorophyll fluorescence

We used the chlorophyll fluorescence parameter *F*_v_/*F*_m_, which provides a measure of the maximum efficiency of photosystem II, as a proxy for drought stress. Water loss inhibits the metabolic processes that are required for photosynthesis leading to a decrease in the *F*_v_/*F*_m_ ratio (Murchie and Lawson, 2013). Each cosm was placed in the centre of a CF Imager (Technologica, Colchester, UK) after a minimum of 20 min dark-adaption period. Focus and aperture size were adjusted for each sample separately as indicated by the operating software, and de-noised in the ‘map’ image to remove non-*Sphagnum* pixels as described in the manufacturer’s protocol. Minimum fluorescence (*F*_o_) was measured using low intensity lighting. Maximum fluorescence (*F*_m_) was measured using a saturating pulse of 6843 µmol m^−2^ s^−1^ photon flux density of photosynthetically active radiation, after which *F*_v_/*F*_m_ was calculated using the formula (*F*_m_–*F*_o_)/*F*_m_. In order to determine how chlorophyll fluorescence values change in the different parts of the *Sphagnum* stem as it dries, areas containing the different zones (capitula, live stem, senescing stem) were manually selected and *F*_v_/*F*_m_ calculated for these areas.

### Relative water content

RWC was calculated for each plate by weighing fresh four capitula and then allowing them to completely dry in an oven at 80 °C for at least 24 h before weighing them again (dry weight). RWC was calculated as a difference between fresh and dry weight divided by dry weight and expressed as a percentage relative to the non-droughted material.

### RNA extraction and qPCR

Capitula from the different species were harvested at the different time points as described above and ground to a fine powder in liquid nitrogen; 500 mg was used for ABA analysis and the rest for RNA extraction using the Spectrum Total RNA kit (Sigma) with in-column DNase treatment (Qiagen) following the manufacturer’s instructions. RNA integrity was determined by gel electrophoresis and 500 ng of total RNA was used to prepare cDNA using the SuperScript III First Strand Synthesis System (Thermo Fisher Scientific). Quantitative polymerase chain reactions (qPCRs) were performed using a LightCycler 480 (Roche). Drought responsive genes were selected based on a study (Cuming *et al.*, 2007) in *P. patens* and *Sphagnum* homologues identified using Phytozome (https://phytozome.jgi.doe.gov/). The gene identifiers for both *P. patens* and *Sphagnum* are included in Supplementary Table S1. Specific primers able to amplify the genes in the four different species were designed using Primer 3 (Supplementary Table S1). Typically, 10 ng of cDNA was used in a 20 μl reaction containing 0.25 μM of each primer and 10 μl LightCycler 480 SYBR Green I Master (Roche). Three biological samples per species per time point were used and each reaction was carried out in triplicate. *Sphagnum* EF1α (Sphfalx03G087000) and GAPDH (Sphfalx16G076000) transcripts were used as references (Winnicka and Melosik, 2019; see Supplementary Table S1 for primer sequences). Data were analysed using the LightCycler 480 Software (Roche). The data shown are means ±SD of three biological replicates.

### ABA measurements

Capitula from the different species were harvested at the different time points as described above and ground in liquid nitrogen to a fine powder. For each sample 50–500 mg of tissue was weighed and extraction performed in 70% (v/v) HPLC grade MeOH (Fisher Scientific) with ultra-pure H_2_O (18.2 Ω). The samples were analysed with flow infusion–electro-spray ionization–high resolution mass spectrometry (FI-ESI-MS). The mass spectra were acquired on an Accela (ThermoFinnigan, San Jose, CA, USA) ultra-performance liquid chromatography system coupled to an Exactive Orbitrap (ThermoFinnigan) mass spectrometer. An aliquot of 20 μl was delivered to the ESI source in 70% (v/v) HPLC grade MeOH (Fisher Scientific) with ultra-pure H_2_O (18.2 Ω). The flow rate was 200 μl min^−1^ for the first 1.5 min and 600 μl min^−1^ for the remaining 1.5 min. Both ionization modes were acquired. ABA peak intensities (*m/z* 263.129 [M-H]-) were converted to μg ml^−1^ concentration based on the derivation of a standard curve based on commercially obtained ABA (Sigma-Aldrich, UK) that was assessed by FI-ESI-MS. Values obtained were then adjusted for the sample RWC. RWC values for time point 0 were considered to be 1 and all the other values calculated in relation to that.

### ABA and desiccation treatments

*Sphagnum fallax* plants were placed in cosms as described above at a density of 33 plants per plate, four plates per each of the three ABA treatments and two desiccation regimes, 24 plates in total. The plates were then placed in tubs filled with water, 10 μM ABA, or 50 μM ABA solution so that about two-thirds of the cosm was submerged and left for 24 h. After this time, the liquid was drained out and excess allowed to drain from the plates for 1 h. All the cosms were weighed (starting weight) and chlorophyll fluorescence measured (see above), and this corresponded to time point 0. Plates were again weighed, and imaged every day until each plate reached a value of *F*_v_/*F*_m_ of 0.2. At that point the plate was immediately rehydrated in water overnight (1 d water withdrawal) or left for 4 d before rehydration (4 d water withdrawal) according to what treatment it was part of. Plates were then weighed and chlorophyll fluorescence measured 1 and 2 d after rehydration. Chlorophyll fluorescence recovery was calculated as the ratio of the *F*_v_/*F*_m_ measurement at day 2 after re-watering and the *F*_v_/*F*_m_ measurement on day 0.

### Phylogenetic analysis of ABA signalling genes in *Sphagnum*

For the construction of the phylogenetic trees, we used a dataset of homologues from Arabidopsis (*Arabidopsis thaliana* Araport11, Cheng *et al.*, 2017), *Oriza sativa* (*Oryza sativa* v7.0, Ouyang *et al.*, 2007), *Selaginella moellendorffii* (*Selaginella moellendorffii* v1.0, Banks *et al.*, 2011), *Marchantia polymorpha* (*Marchantia polymorpha* v3.1, Bowman *et al.*, 2017), *Physcomitrium patens* (*Physcomitrium patens* v3.3, Lang *et al.*, 2018), *Sphagnum fallax* (*Sphagnum fallax* v1.1, DOE-JGI, http://phytozome.jgi.doe.gov/), *Sphagnum magellanicum* (*Sphagnum magellanicum* v1.1, DOE-JGI, http://phytozome.jgi.doe.gov/), and *Ceratopteris richardii* (*Ceratopteris richardii* v2.1, DOE-JGI, http://phytozome.jgi.doe.gov/, for ABI3 only). The homologues were obtained with a BLASTP search against the proteome database from Phytozome 13 (https://phytozome-next.jgi.doe.gov; Goodstein *et al.*, 2012). Deduced amino acid sequences were aligned using MAFFT (Katoh *et al.*, 2019). After elimination of all positions of gaps and short sequences manually, conserved amino acid residue were used in calculations for each gene using the maximum likelihood (ML) method and JTT model (Jones *et al.*, 1992) to construct a ML tree in MEGA-X (Kumar *et al.*, 2018). Statistical support for internal branches by bootstrap analyses (Felsenstein, 1985) was calculated using 1000 replications. All sequences were obtained using the Arabidopsis homologues as query. To build the tree for ABI3 proteins, a significant similarity E-value<1e−10 was used, amino acid sequences lacking conserved B1, B2, and B3 regions (Marella *et al.*, 2006) were deleted from the alignment, and 191 amino acid residues were used to calculate evolution distances for 18 genes. For SnRK2 an E-value<1e−63 was used and 300 amino acid residues were used to calculate evolution distances for 35 genes. For PYR1 the E-value was <1e−11 and 129 amino acid residues were used to calculate evolution distances for 48 genes. For ABI1 an E-value <1e−35 was used and 234 amino acid residues were used to calculate evolution distances for 53 genes. For ABI5, an E-value <1e−8 was used, the amino acid sequences lacking a conserved domain (bZIP_plant_BZIP46: cd14707) were deleted from the alignment, and 53 amino acid residues were used to calculate evolution distances for 47 genes.

### Sphagnum ABI3 gene identification and cloning

*Sphagnum* ABI3 homologues were identified using the *P. patens* ABI3C protein sequence (Pp3c4_7328V3.1) to interrogate the translated *Sphagnum fallax* database in Phytozome (https://phytozome.jgi.doe.gov/; Goodstein *et al.*, 2012). Specific primers were designed for the selected candidate genes and used to amplify the corresponding transcripts from *Sphagnum* cDNA (Supplementary Table S1). The transcript from Sphfalx01G102500 (which we called *SphABI3-16*) was the most abundant under our conditions and the full length cDNA cloned into pDONR207 using Gateway technology (Thermo Fisher Scientific). *PpABI3A* coding region was amplified with specific primers (Supplementary Table S1) from *P. patens* cDNA and then cloned into pENTR/D-TOPO (Thermo Fisher Scientific) according to the manufacturer’s instructions. *SphABI3-16*, and *PpABI3A* full length cDNA sequences were transferred to pMN601 expression vector (Yoshida *et al.*, 2019) under the control of the EF1α promoter by LR reaction (Gateway, Thermo Fisher Scientific).

### *Physcomitrium patens* transformation and ABA sensitivity assays

*SphABI3-16* and *PpABI3A* in pMN601 and pMN601 empty vector were used to transform the *P. patens Δabi3* strain (Khandelwal *et al.*, 2010) by the polyethylene glycol-mediated method as previously described (Nishiyama *et al.*, 2000). Colonies carrying a successful insert were selected on BCDAT plates containing 75 μg ml^−1^ nourseothricin (NTC, Jena Bioscience). The presence of the insert, the targeting to the *PTA-1* locus and the presence of single and multiple insertions were detected by PCR using specific primers as detailed in Supplementary Fig. S1 and Supplementary Table S1. Lines carrying the transgene were selected and transgene expression confirmed by qPCR using *SphABI3-16* specific primers (Supplementary Table S1). For the colony growth in ABA medium, 10 single leaves of each of the different genotypes were removed from young gametophytes and transferred to both BCD medium and BCD medium supplemented with 25 μM ABA and incubated at 25 °C for 32 d. After this time, plates were scanned and colony diameter measured in ImageJ (https://imagej.nih.gov/). Percentage of growth in the ABA medium was calculated in relation to colonies grown in BCD medium alone. For the ABA protonemal growth assays, an equal amount of material for each genotype was homogenized in sterile water and used to inoculate BCDAT plates overlaid with cellophane disks (3 ml per plate) and allowed to grow for 7 d. After this time, cellophane disks were then transferred to BCD plates and BCD plates supplemented with 25 μM ABA (three replicates per genotype, per treatment) and grown for a further 14 d. After this time, plates where imaged in colour using a flatbed scanner at a resolution of 300 dpi using a white background. For each image, the red, green, and blue channels were separated and binary masks were created in R (version 3.5.2) from the RGB tiff image. The binary masks were reapplied to the original image to create a pair of images with green-dominant-pixels and red-dominant pixels respectively. Green pixels were counted using the filters green:blue>1.5 and green:red>1.05 and red pixels were counted using the filters green:blue>1.5 and red:green>1.05. The percentage of green-dominant pixels in the treatment compared with the control was calculated for each line. The presence of red-dominant pixels was used as a proxy for stress in the treatment plates.

## Results

### Differential responses of *Sphagnum* species to controlled desiccation

**Fig. 2.**
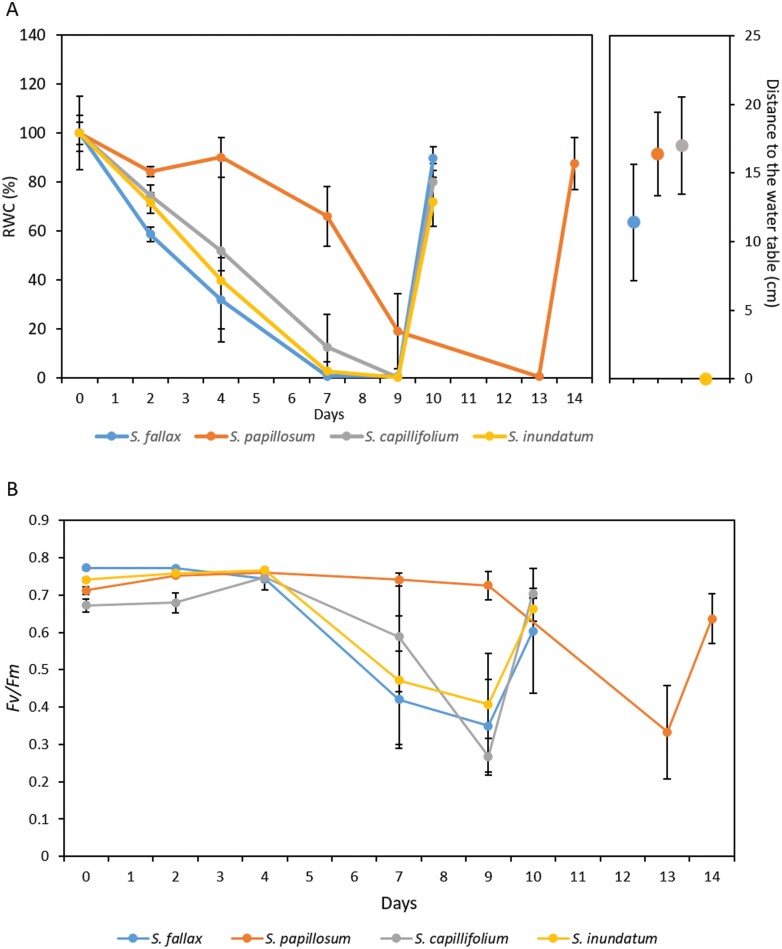
The effect of desiccation on relative water content (RWC) and chlorophyll fluorescence in the four different *Sphagnum* species. Numbers on the *x*-axis indicate days after water withdrawal. *Sphagnum fallax*, *S. capillifolium*, and *S. inundatum* were re-watered on day 9 and *S. papillosum* on day 13. (A) Changes in RWC across the water withdrawal period for the different species. RWC was set to 100% for time zero and the percentage for the other time points calculated in relation to that time point. Data represent means ±SD of four replicate plates. The right-hand side panel shows the water table height; mean values ±SD for four measurements for the four species at the time of harvest. (B) The effect of desiccation on chlorophyll fluorescence (measured as *F*_v_/*F*_m_, see ‘Materials and methods’) over time in the four different species as indicated. Data represent means ±SD of four replicate plates.

To establish if *Sphagnum* species show differential desiccation responses related to their position within the habitat, we chose four local species, representing the four major subgenera and with different distributions across the water table. *Sphagnum fallax* (subgenus *Cuspidata*) is found in lawns and hummocks, *S. papillosum* (subgenus *Sphagnum*) and *S. capillifolium* (subgenus *Acutifolia*) are predominantly hummock species, while *S. inundatum* (subgenus *Subsecunda*) is a typical hollow/pool species (Fig. 1A). All four species are present in the bog at Pen y Garn (van de Koot *et al.*, 2021) at different distances from the water table. *Sphagnum capillifolium* grows further away from the water table while *S. inundatum* is found primarily in ponds. *Sphagnum fallax* and *S. papillosum* occur at similar distances from the water table (Fig. 1A). These four *Sphagnum* species were collected from the site (Fig. 1A top row images) and subjected to a desiccation treatment in cosms, under greenhouse conditions (Fig. 1A bottom row, B). Besides occupying different microhabitats we noted marked morphological differences between the species, including colony density, which may affect responses to desiccation treatments (Fig. 1C). To take this into consideration, the average stem density in the field was determined for each species and used when planting the cosms. Cosms were weighed and imaged regularly over the water withdrawal period. Fresh weight loss and relative water content was calculated for each time point and the stress caused by water withdrawal assessed by measuring the photosynthetic activity recovery of PSII by calculating the *F*_v_/*F*_m_ parameter in dark adapted samples (Maxwell and Johnson, 2000). *Sphagnum fallax*, *S. capillifolium*, and *S. inundatum* showed similar rates of fresh weight loss and, 9 d after water withdrawal, plant weight was down to less than 10% of the original weight (Supplementary Fig. S2). *Sphagnum papillosum* showed much slower fresh weight loss and did not reach the same stage of desiccation until 13 d after water withdrawal (Supplementary Fig. S1). *Sphagnum*’s ability to store water has been attributed to anatomical features such as the size of capitula and the shape of the branches as well as the shape and number of specialized water-storage hyaline cells (Bengtsson *et al.*, 2020). To take this into account, we calculated the RWC for each sample. As seen with the fresh weight loss, *S. fallax* and *S. inundatum* showed a rapid decrease in RWC and by 7 d after water withdrawal the RWC of the capitula was near zero, while *S. capillifolium* showed a slightly slower decrease (Fig. 2A). *Sphagnum papillosum* again showed a slow decrease in RWC across time and only reaching a similar point to the other species at day 13 (Fig. 2A). While the slower decrease in *S. papillosum* and to a lesser extent in *S. capillifolium* can be correlated with their position in relation to the water table, the same is not true for *S. fallax* and *S. inundatum* as they show similar rates of fresh weight loss despite being collected at different water table distances (Fig. 2A). We next looked at the effect of desiccation on the photosynthetic activity of the different species by calculating the *F*_v_/*F*_m_ ratio (see ‘Materials and methods’). Within the first 4 d after water withdrawal, the *F*_v_/*F*_m_ ratio stayed constant or even showed a small increase in some of the species (Fig. 2B). This increase in photosynthetic activity might be due to the reduction of the excess surface water allowing improved gas exchange. From day 4 onwards, the *F*_v_/*F*_m_ ratio started to decrease, and as observed with the RWC, this decrease was more pronounced in *S. fallax* and *S. inundatum* and slightly slower in *S. capillifolium* (Fig. 2B). By day 9, these three species showed *F*_v_/*F*_m_ values between 0.2 and 0.4. In *S. papillosum*, the ratio *F*_v_/*F*_m_ decreased more slowly and only reached a value of 0.3 at day 13 (Fig. 2B). At *F*_v_/*F*_m_ values between 0.2 and 0.4, full recovery was still possible when plates were re-watered (Fig. 2B). We also measured *F*_v_/*F*_m_ recovery after re-watering when compared with the starting *F*_v_/*F*_m_ ratio (Supplementary Fig. S3). *Sphagnum capillifolium*, present at higher water table distances, fully recovered while *S. inundatum* and *S. papillosum* had recovery rates of around 90%. *Sphagnum fallax* showed high variation in recovery between replicates with three out of four replicates making full recovery and one not recovering (Supplementary Fig. S3).

In addition to whole plant responses, we also looked at how different parts of the *Sphagnum* plant responded to desiccation. For this, we divided the chlorophyll fluorescence images into three regions, one encompassing the capitulum (top), the other the middle section of the stem (middle), and the last the basal senescing stem region (base), and calculated the *F*_v_/*F*_m_ ratio for these regions across the water withdrawal period. The general changes in *F*_v_/*F*_m_ of the three regions for the four species mirrored what was seen for the whole plant but there were differences in the rate of change (Supplementary Fig. S4). While the three regions showed similar starting values for *F*_v_/*F*_m_, as expected the top region showed a faster decrease in *F*_v_/*F*_m_ suggesting a faster drying rate (Supplementary Fig. 4A) while the base, less exposed to air flow, dried more slowly (Supplementary Fig. S4C).

### Induction of drought-responsive genes in the different *Sphagnum* species

**Fig. 3.**
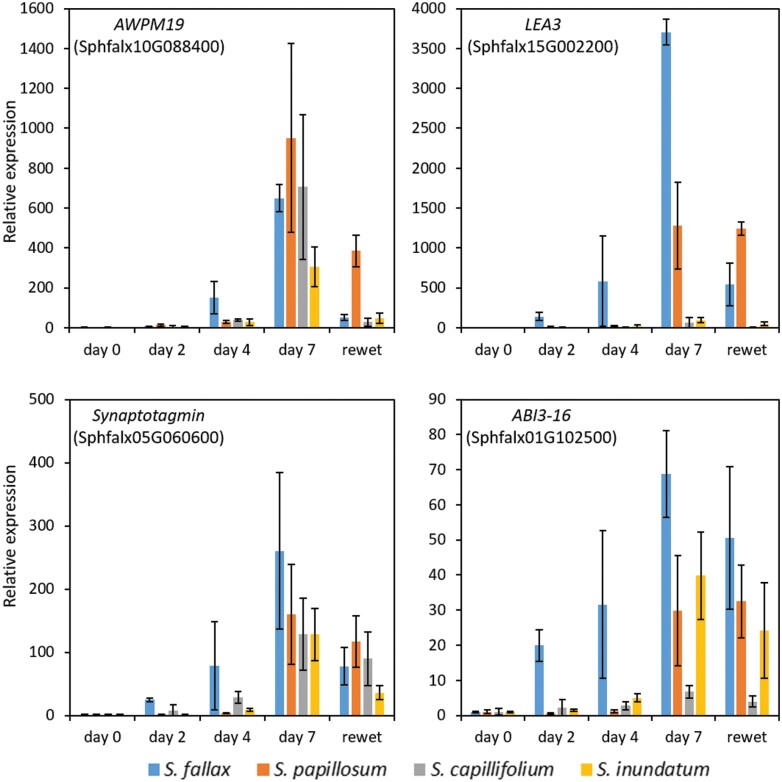
Analysis of drought-responsive gene expression in the four different *Sphagnum* species. Plants in cosms were droughted for 7 d and then re-watered. Samples for RNA extraction were collected at 0, 2, 4, and 7 d and from re-watered material (0, 4, 7, 9 d and re-watered for *S. papillosum*). Expression of *AWPM19* (Sphfalx10G088400), *LEA3* (Sphfalx15G002200), *Synaptotagmin* (Sphfalx05G060600), and *ABI3-16* (Sphfalx01G102500) was determined by qPCR using *EF1α* (Sphfalx03G087000) and *GAPDH* (Sphfalx16G076000) as reference genes as indicated over each graph. Each sample was done in triplicate and data represent means ±SD of three biological replicates. Expression at day 0 was set to 1 and all the other expression values calculated in relation to day 0.

After determining that there were indeed differences in physiological responses to desiccation, we next asked if these changes are accompanied by changes in the expression of drought-responsive genes. For this we selected four genes known to be strongly up-regulated upon drought in the moss *P. patens* (Cuming *et al.*, 2007) and identified the *Sphagnum* homologues (Supplementary Table S1). *AWPM19* encodes a drought-induced membrane protein that in rice promotes ABA influx into the cell (Yao *et al.*, 2018); late embryogenesis abundant proteins group 3 (LEAs group 3) are also induced in response to desiccation and are important during seed maturation (Battaglia *et al.*, 2008); synaptotagmins maintain membrane integrity under stress conditions (Schapire *et al.*, 2008) and ABI3 is a central component of the ABA-induced desiccation tolerance in the moss *P. patens* (Khandelwal *et al.*, 2010). We observed increased expression of all genes as desiccation progressed but the magnitude of the change was different for each species (Fig. 3). *Sphagnum fallax* and *S. papillosum* showed larger increases in expression of the genes, with expression peaking at day 7, while in *S. capillifolium* and *S. inundatum* the relative increases were more modest (Fig. 3). Interestingly, these two species showed higher basal levels of expression in untreated material (Supplementary Fig. S5). We observed a marked decrease in drought-responsive gene expression 24 h after re-watering except for *S. papillosum* where expression was maintained at high levels (Fig. 3). It is worth noting the high variability between the three biological samples seen in the large SD observed, especially at later time points, which probably reflect different drying rates for each cosm.

### ABA mediates desiccation responses in *Sphagnum*

**Fig. 4.**
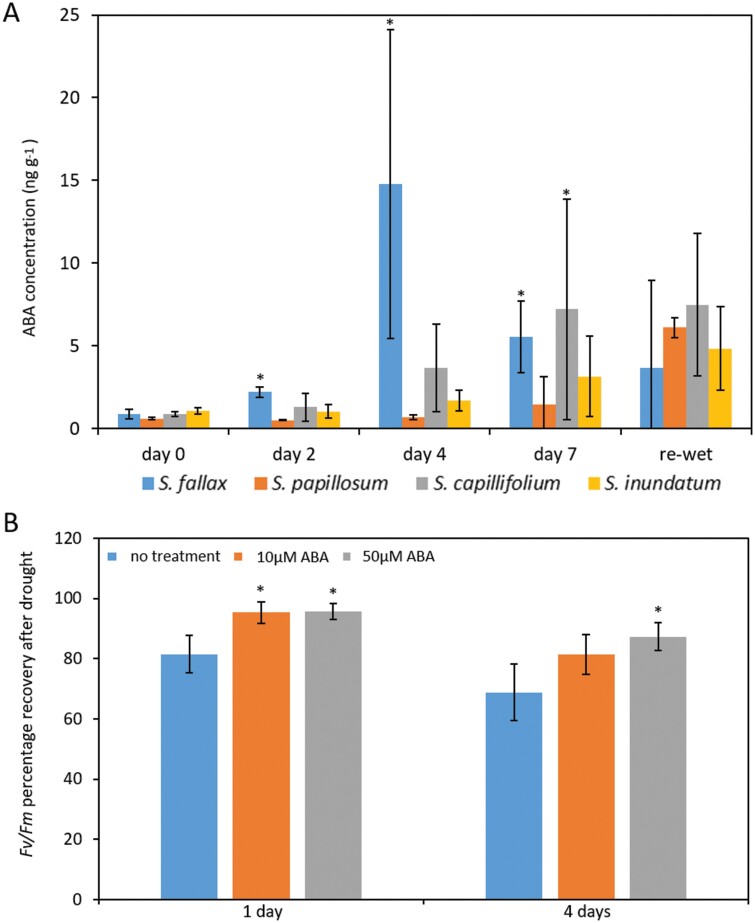
ABA signalling pathways are activated upon desiccation in *Sphagnum*. (A) Desiccation increases ABA in *Sphagnum*. Capitula from the different species were harvested at 0, 2, 4, 7 d after drought and from re-watered samples. ABA levels were determined by GC-MS. ABA concentrations were then adjusted in relation to the relative water content of each sample. Data represent means ±SE for three biological replicates. (B) ABA pre-treatment improves desiccation tolerance in *Sphagnum*. *Sphagnum fallax* plants were desiccated until *F*_v_/*F*_m_ value reached 0.2 and kept at this stage for 1 or 4 d before re-watering as indicated. The percentage *F*_v_/*F*_m_ recovery was calculated at day 2 after re-watering in relation to the initial *F*_v_/*F*_m_ values. Data represent means ±SD for four replicate plates. *Significantly different from the untreated plants, *P*<0.05.

It is well established that ABA is a central regulator of drought responses in plants and that ABA production increases upon drought. Despite this, not much is known about the role of ABA in desiccation tolerance in *Sphagnum*. We measured ABA concentration per gram fresh weight, across the water withdrawal period for all species. As the amount of water held by the plants varies drastically between samples as they dry, we adjusted the values using the RWC for each sample (see ‘Materials and methods’). We detected a low concentration of ABA in well-watered material (day 0) for all four species but ABA levels generally increased as drought progressed, peaking around day 4 (Fig. 4A), preceding the peak of ABA-dependent gene expression at day 7 (Fig. 3). Similar to what was observed with ABA-induced gene expression, the increase was more pronounced in *S. fallax* (Fig. 4A). We also observed significant increases in endogenous ABA for *S. capillifolium* at day 7, while ABA levels increased slightly but not significantly in *S. papillosum* and *S. inundatum* (Fig. 4A). Twenty-four hours after being re-watered, ABA levels had not yet returned to basal levels in the four species (Fig. 4A).

Increases in ABA and ABA-responsive gene expression underpin drought responses, increasing the ability of the plants to tolerate mild drought (Chaves *et al.*, 2003). To determine if this is also the case in *Sphagnum*, we tested the effect of ABA pre-treatment on the desiccation response of *S. fallax* plants in cosms. We used two ABA concentrations, 10 μM and 50 μM, to pre-treat *S. fallax* plants for 24 h before water withdrawal. Desiccation responses were evaluated by measuring photosynthetic performance by calculating the *F*_v_/*F*_m_ ratio. *Sphagnum fallax* plants were allowed to dry until *F*_v_/*F*_m_ reached 0.2 and kept at this value (i) for 1 d and then re-watered or (ii) for 4 d and then re-watered. After re-watering, *F*_v_/*F*_m_ was again measured and the percentage recovery (*F*_v_/*F*_m_ after re-watering/starting *F*_v_/*F*_m_×100) was calculated for all treatments. Pre-treatment with either 10 μM or 50 μM ABA increased *F*_v_/*F*_m_ recovery after 1 d at *F*_v_/*F*_m_ of 0.2, while at the harsher treatment of 4 d at *F*_v_/*F*_m_ of 0.2, only 50μM ABA significantly improved recovery (Fig. 4B).

Taken together, these data suggest that ABA is an important component of desiccation responses in *Sphagnum*.

### Many genes involved in ABA signalling pathways are conserved in *Sphagnum*

Many of the ABA signalling components from perception to signal transduction have been identified in the model plant Arabidopsis and found to be conserved across many other species (Cai *et al.*, 2017; Sun *et al.*, 2020). Querying of the available *Sphagnum* genome sequences (*S. fallax* and *S. magellanicum*) showed that these components are also present in *Sphagnum* (Supplementary Figs S6–S8). All the core ABA signalling proteins, PYR/RYL (ABA receptors; Supplementary Fig. S6), SnRK2 (ABA-signalling regulators, Supplementary Fig. S7), and ABI5s (transcription factors phosphorylated by activated SnRK2, Supplementary Fig. S8) were found in the *Sphagnum* genome. In particular, the phylogenetic trees of PYR/RYC and SnRK2 indicate that bryophyte species have fewer PYR/RYC and SnRK2 genes than vascular plants (Supplementary Fig. S6, S7). Surprisingly, while homologues of the clade F of protein phosphatases (PP2Cs) are present in *Sphagnum*, we were not able to find any homologues for clade A (Supplementary Fig. S9). This clade of PP2Cs is found in other bryophytes and includes Arabidopsis ABI1/2, HAB1/2, and AHG1/3 that together with PYR act as co-receptors for ABA (Nishimura *et al.*, 2010).

The plant-specific ABI3 family of transcription factors plays a critical role during seed development desiccation tolerance and is an important regulator of ABA-mediated responses to abiotic stresses (Sakata *et al.*, 2010). In *P. patens*, mutants (*Δabi3*) lacking three of the ABI3 genes (*PpABI3A/B/C*) have increased drought and desiccation tolerance and reduced sensitivity to exogenously applied ABA (Khandelwal *et al.*, 2010; Tan *et al.*, 2017; Zhao *et al.*, 2018).

**Fig. 5.**
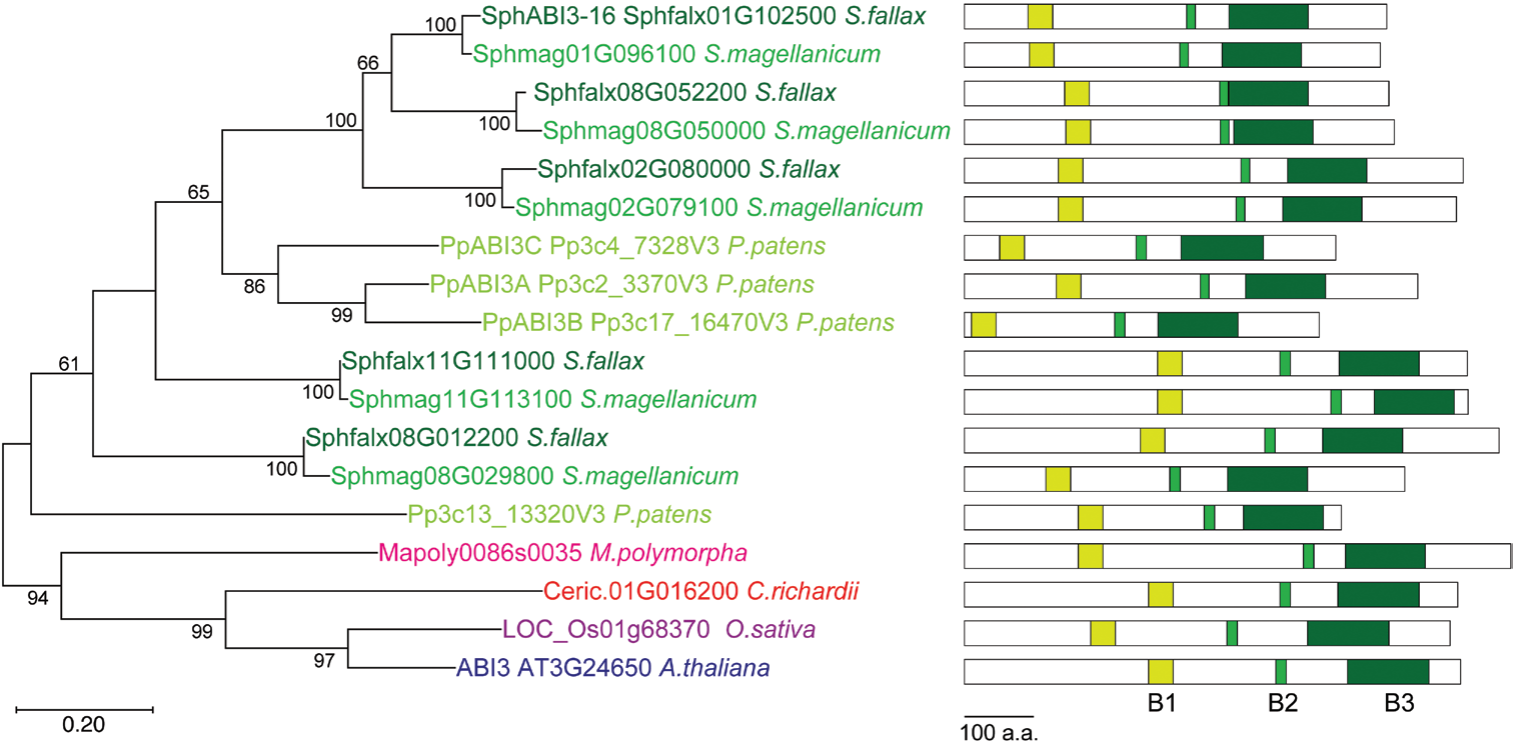
A phylogenetic tree of ABI3 proteins. The tree was constructed with the maximum likelihood method using the JTT-based matrix. The tree with the highest log likelihood is shown. Bootstrap values of >50% are shown on the branches. Horizontal branch length is proportional to the estimated evolutionary distance. Schematic representations of the corresponding protein structure, including the conserved B1–B3 domains indicated by the green boxes, are also shown.

**Fig. 6.**
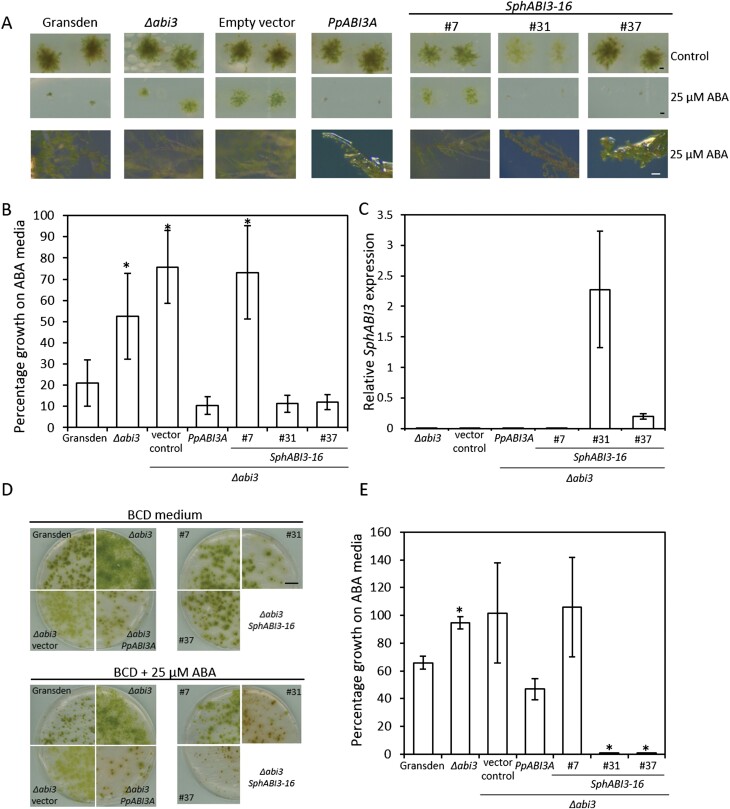
*Sphagnum* ABI3 rescues the phenotype of the *Δabi3* mutant in *P. patens*. (A) Colony growth in wild type *P. patens* (Gransden), *Δabi3* mutant, and *Δabi3* mutant transformed with all the different potential rescue constructs as indicated. Single leaves were grown in BCD medium (top row) or BCD medium supplemented with 25 μM ABA (middle row) for 32 d. Scale bar: 500μm. Bottom row shows detail of the colony structure of the plants grown in the ABA medium (scale bar: 100 μm). (B) Relative colony growth in ABA medium for wild type (Gransden), *Δabi3* mutant, and the rescue transformants as shown above. After 32 d growth, colony diameter was measured and the percentage of growth in the ABA-containing medium was calculated in relation to the media without ABA. Data show means ±SD of 10 colonies. *Significantly different from wild type, *P*<0.001. (C) Increased *ABI3* expression in the transformed *Δabi3* mutant was confirmed by qPCR. Data show means ±SD of three replicates. Expression of *P. patens EF1α* was used as a reference. (D, E). Protonemal growth of wild type (Gransden), *Δabi3* mutant and the rescue transformants in BCD medium or BCD medium supplemented with 25 μM ABA as indicated. (D) Representative images of each genotype after 14 d growth. (E) Quantification of protonemal growth. After 14 d growth, plates were scanned and growth (total green pixels in the image) was calculated for each plate. The percentage of growth in the ABA containing medium was calculated in relation to the medium without ABA. Data show means ±SE of three plates per genotype. *Significantly different from wild type, *P*<0.001.

In order to determine if these functions are conserved in ABI3 proteins from *Sphagnum* moss, we cloned one of the five *Sphagnum* ABI3 homologues (Fig. 5; SphABI3-16 (Sphfalx01G102500)) and asked if it could rescue the *Δabi3* phenotype. When grown on medium supplemented with 25 µM ABA for 32 d, wild type *P. patens* showed around 20% of the growth in the ABA-free medium while the *Δabi3* mutant showed a growth of around 50% (Fig. 6A, B). This increased growth was maintained when the *Δabi3* mutant was transformed with an empty vector construct, but growth returned to wild type levels when the *Δabi3* mutant was transformed with the endogenous *PpABI3A* gene (Fig. 6A, B). When the *Δabi3* mutant was transformed with *SphABI3-16*, growth in ABA medium was also inhibited, returning to wild type levels in lines #31 and #37 (Fig. 6A, B). Interestingly, in line #7, growth was indistinguishable from the untransformed *Δabi3* mutant (Fig. 6A, B) but, despite carrying the *SphABI3-16* construct, we could not detect expression in this line (Fig. 6C). As the transgene in this line is not found at the *PTA-1* locus (Supplementary Fig. S1), the lack of expression might be due to where the transgene was inserted within the genome.

Exogenous ABA application is also known to affect protonemal growth and choronemal cell morphology in *P. patens*. Protonemal growth was inhibited when wild type *P. patens* was grown on medium containing 25 μM ABA (Fig. 6D, E). Consistent with having decreased sensitivity to ABA, this growth inhibition was not observed in the *Δabi3* mutant. Expression of *PpABI3A* in the *Δabi3* mutant background recovered the wild type phenotype (Fig. 6D, E). Expression of *SphABI3-16* in the *Δabi3* mutant background not only restored growth sensitivity to ABA, but also resulted in the protonemal growth becoming oversensitive to exogenous ABA application (Fig. 6D, E). Interestingly, the *Δabi3* lines expressing either *PpABI3A* or *SphABI3-16* when grown on ABA medium also produced increased amount of red pigments suggesting increased stress levels in these plants (Supplementary Fig. S10). Another effect of ABA application is the increased branching of chloronemal cells. As expected from the reduced sensitivity of the *Δabi3* mutant, we observed reduced chloronemal branching when this line was grown on ABA-containing medium (Fig. 6A, bottom row). Consistent with the results described above, the *Δabi3* mutant transformed with either the endogenous *PpABI3A* or *SphABI3-16* showed wild-type like phenotype of increased branching (Fig. 6A, bottom row).

These data indicate that *Sphagnum* contains an ABA signalling system functionally equivalent to that of other mosses and higher plants, further supporting a role for ABA during desiccation responses in *Sphagnum*.

## Discussion

By using a comprehensive physiological and molecular approach, we show that different *Sphagnum* species respond differently to a controlled induced desiccation, but, surprisingly, this is not always correlated with their position relative to the water table.

*Sphagnum* mosses have been considered to be desiccation-avoiding rather than tolerant (Clymo, 1973; Clymo and Hayward, 1982). In this scenario, they have evolved systems to store large amounts of water with 90% of all water stored in extracellular spaces and 10% in hyaline cells (Clymo and Hayward, 1982). Detached *Sphagnum* capitula have been shown to have a water content of 700–1200% relative to plant weight for optimal photosynthesis (Bengtsson *et al.*, 2016), high values as compared with other bryophytes and most other land plants. Of the four species that we used for our studies, *S. inundatum* showed the highest capitula water content (2498 ± 181%), followed by *S. papillosum* (2121 ± 205%) and *S. fallax* and *S. capillifolium* having similar water content values (1458 ± 107% and 1441 ± 217%). Unexpectedly these values do not correlate with their position along the water table (Fig. 2A) but are probably a reflection of plant morphology (Fig. 1). Both *S. papillosum* and *S. inundatum* have larger capitula than the other two species and have longer more densely packed branches, which are able to store larger amounts of water (Fig. 1). After water withdrawal, all species except *S. papillosum* lost water very quickly, reaching a RWC of 10–20% within a week. The slower fresh weight loss in *S. papillosum* is probably due to a combination of larger capitula and longer branches and the densely packed canopy they form. On the other hand, while *S. inundatum* plants also have large capitula, they are found in pools at low plant density and this may explain the fast fresh weight loss. A recent study by (Bengtsson *et al.*, 2020) showed that there is a correlation between anatomical traits such as leaf width and hyaline cell pore size and water retention. The authors also showed similar relationships between RWC and *F*_v_/*F*_m_ for *S. fallax* and *S. papillosum* species in common with our study. Interestingly, their pool species, *S. cuspidatum*, also showed high initial RWC, as we observed in *S. inundatum* (Bengtsson *et al.*, 2020). Although previous studies (Schipperges and Rydin, 1998) suggest that at RWC levels lower than 10–20%, *Sphagnum* plants do not recover, we found that even at RWC values near zero, recovery after re-watering was nearly 100% (Fig. 2A). It should be noted that we found that longer periods at RWC<10% did affect survival in all species.

Our data show that the initial rapid fresh weight loss does not have an immediate impact on the physiological status as determined by the *F*_v_/*F*_m_ ratio, which measures the quantum efficiency of photosystem II and gives an indirect measure of plant stress (Fig. 2B). For *S. fallax*, S*. capillifolium*, and *S. inundatum*, the *F*_v_/*F*_m_ ratio was stable for the first 4–5 d suggesting that the plant was not stressed at RWC of around 50%. The first physiological signs of stress appeared between day 5 and day 7 after water withdrawal. During this period, the RWC fell to less than 20% and there was a sharp decrease in the *F*_v_/*F*_m_ ratio to values between 0.4 and 0.6, accompanied by the induction of the expression of drought-related genes (Fig. 3). At day 9, the RWC neared zero and the *F*_v_/*F*_m_ ratio decreased to around 0.2. For *S. papillosum* on the other hand, this was not attained until day 13, again underscoring the high water holding capacity of this species. As early as 24 h after re-watering, both the RWC and the *F*_v_/*F*_m_ ratio recovered to levels close to the starting point, suggesting that *Sphagnum* is able to tolerate mild desiccation without significant plant damage. While some studies describe similar levels of recovery, others report very poor recovery after desiccation (Wagner and Titus, 1984; Gerdol *et al.*, 1996; Hájek and Beckett, 2008; Hájek and Vicherová, 2014). One possible factor in these differences is the levels of hardening that the plants were subjected to before the experiment. The plants we used had been naturally exposed to varied degrees of desiccation and rehydration in the field and we did not allow for any greenhouse adaptation time. They may have developed a degree of hardening that was impacting on the observed desiccation tolerance. In agreement with some recent studies, we did not observe significant differences in desiccation tolerance between hummock and hollow species (Wagner and Titus, 1984; Schipperges and Rydin, 1998; Oliver *et al.*, 2005; Proctor *et al.*, 2007; Hájek and Beckett, 2008; Hájek and Vicherová, 2014; Bengtsson *et al.*, 2020).

The molecular signatures of drought responses seem to be conserved in *Sphagnum* as many of the genes found to be up-regulated by drought in other species are also up-regulated in drying *Sphagnum* (Fig. 3), and together with the observed increases in ABA, this suggests the existence of an inducible desiccation response pathway in *Sphagnum*.

### ABA as an important component of desiccation responses in *Sphagnum*

Evolutionary analysis of ABA signalling pathways suggest that ABA has been used ancestrally as a stress hormone in land plants and that it might have evolved to enable drought and desiccation tolerance in the new land environment (Wang *et al.*, 2015; Cuming, 2019; Sun *et al.*, 2020). Many of the ABA synthesis, perception, and signalling components have been identified in a range of land plants and their functions seem to be broadly maintained (Guillory and Bonhomme, 2021). In the mosses *P. patens* and *Funaria*, drought has been shown to increase endogenous ABA concentrations (Werner *et al.*, 1991; Knight *et al.*, 1995). ABA pre-treatment has been shown to increase drought tolerance in several crop species (Lu *et al.*, 2009; An *et al.*, 2014; Changning *et al.*, 2014; Liting *et al.*, 2015; Brito *et al.*, 2020; Skowron and Trojak, 2021) and also in mosses, including one study in *Sphagnum* (Wagner and Titus, 1984; Beckett *et al.*, 2000; Hájek and Beckett, 2008; Khandelwal *et al.*, 2010). We also observed an increase of endogenous ABA during desiccation in *Sphagnum*. ABA levels were at the highest at day 4 (Fig. 4), preceding the peak of ABA-induced gene expression observed at day 7 (Fig. 3). ABA levels did not increase after day 4, in line with what was observed in other species (Scholz *et al.*, 2015). Notably, 24 h after the plants were re-watered, we observed decreased gene expression but levels of ABA were still high, suggesting that it takes longer for ABA levels to return to normal in re-watered *Sphagnum* plants. In *S. fallax*, the most desiccation sensitive of the species used, the increases in ABA levels were the highest while more modest increases were observed in *S. capillifolium*. In the most desiccation resistant species, *S. papillosum*, levels of ABA were lowest, and possibly sampling at later time points would be necessary to detect larger increases in ABA. In *S. inundatum*, ABA levels stayed constant at low levels and this was also the case for drought responsive gene expression (Figs 3, 4). Being a hollow species, one would expect that *S. inundatum* would need to quickly respond to the lack of water, and it has been proposed that it does not possess the same desiccation avoidance mechanisms as hummock species (Mazziotta *et al.*, 2019), which may have adapted by evolving the faster ABA-dependent pathways. Our data suggest that this may not be the case and *S. inundatum* relies on other mechanisms for desiccation tolerance such as the proposed resource acquisition (Mazziotta *et al.*, 2019). Alternatively, as the basal level of drought-responsive gene expression is high in this species when compared with the others (Supplementary Fig. S5), it is possible that some aspects of the desiccation response may be constitutively activated in this species.

### ABA-mediated drought signalling is conserved in *Sphagnum*

*Sphagnum* species also merit attention in the context of the plant’s transition from aquatic to terrestrial life. A critical step in the water to land transition was the acquisition of desiccation tolerance and, although sphagna are thought to be mainly desiccation avoidant, our data suggest that they have a basal level of desiccation tolerance and this involves, at least in part, ABA. We found that the main components of ABA responses are conserved in *Sphagnum* with the notable exception of clade A PPC2As that include the ABA co-receptor ABI1 (Nishimura *et al.*, 2010; Bhaskara *et al.*, 2019). The fact that we were not able to find ABI1 homologues in the *Sphagnum* genome could be due to the homology thresholds we used, although this is unlikely as we were able to find homologues in *P. patens* and *M. polymorpha*. Another possibility is that these genes are not correctly annotated in the *Sphagnum* genome versions that we used to build our phylogenies (*S. fallax* v1.1 and *S. magellanicum* v1.1). Otherwise, the prospect of ABI1 homologues not being present in *Sphagnum* raises the interesting possibility that *Sphagnum* has co-opted other proteins, maybe PP2Cs from other clades, for example clade F that seems to have expanded in *Sphagnum* (Supplementary Fig. S9), as ABA co-receptors.

Our data also show that the function of ABA signalling components is conserved in *Sphagnum* as demonstrated by the ABI3 protein. In tracheophytes, ABI3 was first identified as a seed specific transcriptional activator (Finkelstein and Somerville, 1990). While its role during seed development remains best documented, ABI3 has been found to be important in other vegetative tissues where it controls developmental transitions and stress responses (Rohde *et al.*, 2000; Bedi *et al.*, 2016). Its functions extend outside seeded plants and may have even diversified in mosses where we find an expansion of the number of ABI3 proteins (five in *Sphagnum fallax* and four in *P. patens* compared with one in Arabidopsis and one in rice; Fig. 5). In *P. patens*, loss of function mutants in three of the *ABI3* genes show decreased ABA sensitivity and are more sensitive to desiccation and other stresses (Marella *et al.*, 2006; Takenaka *et al.*, 2007; Khandelwal *et al.*, 2010; Tan *et al.*, 2017; Zhao *et al.*, 2018). We found that heterologous expression of the *Sphagnum* ABI3 orthologue rescued the phenotype of loss of function *Δabi3* mutant in *P. patens* (Fig. 6). This is the first time that an ABA-pathway gene from *Sphagnum* has been functionally characterized and has several implications. The first is that ABA-mediated responses to stress are conserved in *Sphagnum* as was observed for other mosses (Cuming, 2019; Guillory and Bonhomme, 2021). It will be interesting to determine if the role of ABA in other developmental pathways such as gametophyte development and spore germination is also conserved in *Sphagnum*. In addition, there is evidence that, although there is broader conservation of ABA pathways in other mosses, the molecular and physiological responses as well as the interaction with other hormone pathways are different from the ones observed in vascular plants. Further detailed studies in *Sphagnum* will give better insight into the evolution of ABA signalling pathways (Cuming, 2019; Guillory and Bonhomme, 2021). Secondly, our studies confirm the usefulness of using *P. patens* as a heterologous functional expression system for species without an established transformation protocol (Rensing *et al.*, 2020). The ease of transformation and the existence of well-characterized mutants in many of the developmental and stress signalling pathways in *Physcomitrium* are valuable resources to test the functionality of other *Sphagnum* genes. Lastly, our data support earlier observations that *Sphagnum* is able to tolerate significant levels of dehydration without losing photosynthetic capacity, and this should be taken into consideration when modelling the global responses of peatlands to changes in climate patterns especially precipitation (Wagner and Titus, 1984; Schipperges and Rydin, 1998; Proctor, 2000; Oliver *et al.*, 2005; Proctor *et al.*, 2007; Dise, 2009; Hájek and Vicherová, 2014; Cruz de Carvalho *et al.*, 2019; Rastogi *et al.*, 2020).

## Supplementary data

The following supplementary data are available at *JXB* online.

Fig. S1. Targeting of the *PpABI3A* and *SphABI3-16* expression constructs.

Fig. S2. Percentage fresh weight loss during the desiccation experiment for the four different *Sphagnum* species.

Fig. S3. Chlorophyll fluorescence recovery after desiccation as measured by *F*_v_/*F*_m_.

Fig. S4. Changes of chlorophyll fluorescence upon desiccation in different regions of the *Sphagnum* plants for the four different species.

Fig. S5. Basal expression of drought responsive genes is higher in *S. inundatum*.

Fig. S6. ABA signalling components are present and evolutionarily conserved in *Sphagnum* including the ABA-receptor family PYR1.

Fig. S7. ABA signalling components are present and evolutionarily conserved in *Sphagnum* including SnRK2.

Fig. S8. ABA signalling components are present and evolutionarily conserved in *Sphagnum* including ABI5.

Fig. S9. Clade A PPC2A homologues are absent in *Sphagnum*.

Fig. S10. *Physcomitrium patens* plants with increased expression of ABI3 show signs of stress as measured by the presence of red pixels.

Table S1. List of the primers used in this study.

erac133_suppl_Supplementary_Table_S1_Figures_S1-S10

## Data Availability

All data supporting the findings of this study are available within the paper and within its supplementary materials published online.
