# Supplementary material for: Molecular and physiological responses to desiccation indicate the abscisic acid pathway is conserved in the peat moss, Sphagnum
Source: J Exp Bot. 2022 Apr 6;73(13):4576–91. doi: 10.1093/jxb/erac133 (PMC9291362; doi:10.1093/jxb/erac133)
Supplement: erac133_suppl_Supplementary_Table_S1_Figures_S1-S10 [file erac133_suppl_supplementary_table_s1_figures_s1-s10.pdf]

Table S1. List of the primers used in this study.

Primer Name	Sequence	Purpose	(Physcomitrium patens v3.3, DOE-JGI, https://phytozome-	Gene (Sphagnum fallax v0.5, DOE-JGI, http://phytozome.jgi.doe.gov/info/Sfallax_v0_5)	Gene (Sphagnum fallax v1.1, DOE-JGI, http://phytozome.jgi.doe.gov/info/Sfallax_v1_1)	Reference
EF1α_Fw	TTGCGCTGTTCTCATCATCG	qPCR reference gene		Sphfalx0026s0086	Sphfalx03G087000	doi.org/10.1016/j.ppees.2019.02.004
EF1α_Rv	ATCTGGTCCAAGGCTTCCAA	qPCR reference gene		Sphfalx0026s0086	Sphfalx03G087000	doi.org/10.1016/j.ppees.2019.02.004
GAPDH_Fw	GCAGTGACGTGTCTTCCACT	qPCR reference gene		Sphfalx0068s0064	Sphfalx16G076000	doi.org/10.1016/j.ppees.2019.02.004
GAPDH_Rv	CCAGGCTACAACCTTGACCA	qPCR reference gene		Sphfalx0068s0064	Sphfalx16G076000	doi.org/10.1016/j.ppees.2019.02.004
AWPM19_domain_Fw	GACTTGGGAGAGGGTTGTCT	qPCR	Pp3c4_30620V3.1	Sphfalx0106s0009	Sphfalx10G088400	doi.org/10.1111/j.1469-8137.2007.02187.:
AWPM19_domain_Rv	CGCTCGCTATCAGTGCAATT	qPCR	Pp3c4_30620V3.1	Sphfalx0106s009	Sphfalx10G088400	doi.org/10.1111/j.1469-8137.2007.02187.:
Synaptotagmin_Fw	ATGTGTTGGGTGGGGATCTT	qPCR	Pp3c20_8650V3.1	Sphfalx0162s0043	Sphfalx05G060600	doi.org/10.1111/j.1469-8137.2007.02187.:
Synaptotagmin_Rv	AGTTCTTTGCACGGACAACC	qPCR	Pp3c20_8650V3.1	Sphfalx0162s0043	Sphfalx05G060600	doi.org/10.1111/j.1469-8137.2007.02187.:
LEA_Fw	CGATGTCGCAGTATACGCAG	qPCR	Pp3c12_22320V3	Sphfalx0198s0014	Sphfalx15G002200	doi.org/10.1111/j.1469-8137.2007.02187.:
LEA_Rv	GTGGAATCTGCAGCTTGGTC	qPCR	Pp3c12_22320V3	Sphfalx0198s0014	Sphfalx15G002200	doi.org/10.1111/j.1469-8137.2007.02187.:
SfABI3_16_Fw	TGACAGACATCAGGCCAACT	qPCR		Sphfalx0016s0174	Sphfalx01G102500	
SfABI3_16_Rv	CCCTTCTTTCAGCGTGAGAC	qPCR		Sphfalx0016s0174	Sphfalx01G102500	
SfABI3_16_attB1	GGGGACAAGTTTGTACAAAAAAGCAGGCTCG ATGGCTGGGGGAAGAGGAAAGG	cloning		Sphfalx0016s0174	Sphfalx01G102500	
SfABI3_16_attB2	GGGGACCACTTTGTACAAGAAAGCTGGGTTT CAACTTGCTGGCTCAATCTTTG	cloning		Sphfalx0016s0174	Sphfalx01G102500	
**Primer Name**	**Sequence**	**Purpose**				**Reference**
PpABI3A_F1cacc	CACCATGGTGCTCCTATCGAGTGT	cloning	Pp3c2_3370V3			
PpABI3A_R1	TCATCCTGCGGGCTCGGT	cloning	Pp3c2_3370V3			
PpEF1aFw	AATCATACATTTCACCTCGCC	qPCR reference gene	Pp3c2_10310V3			doi: 10.1371/journal.pone.0070998
PpEF1aRv	GATCAGTGGGTAGAAGTGAC	qPCR reference gene	Pp3c2_10310V3			doi: 10.1371/journal.pone.0070998
pTA1cF1	TGAGAAGCCACAACTTTTGGAA	insertion check				
pTA1-3F1	TACAAACCTGTGGACCGCCACGATGCGGT	insertion check				
pTA1cR1	TGCACCCGCTTAAGATGTTC	insertion check				
PpEF1aR1	AATTGATATTGCATGTGTTTTGA	insertion check				
n-F2	AGGAGGAAGACAAGGAAGGATAAGGTTGCA	insertion check				
nat1-atg	ATCACCACTCTTGACGACACGGCT	insertion check				
nat1-tga	GGGGCAGGGCATGCTCATGTA	insertion check				

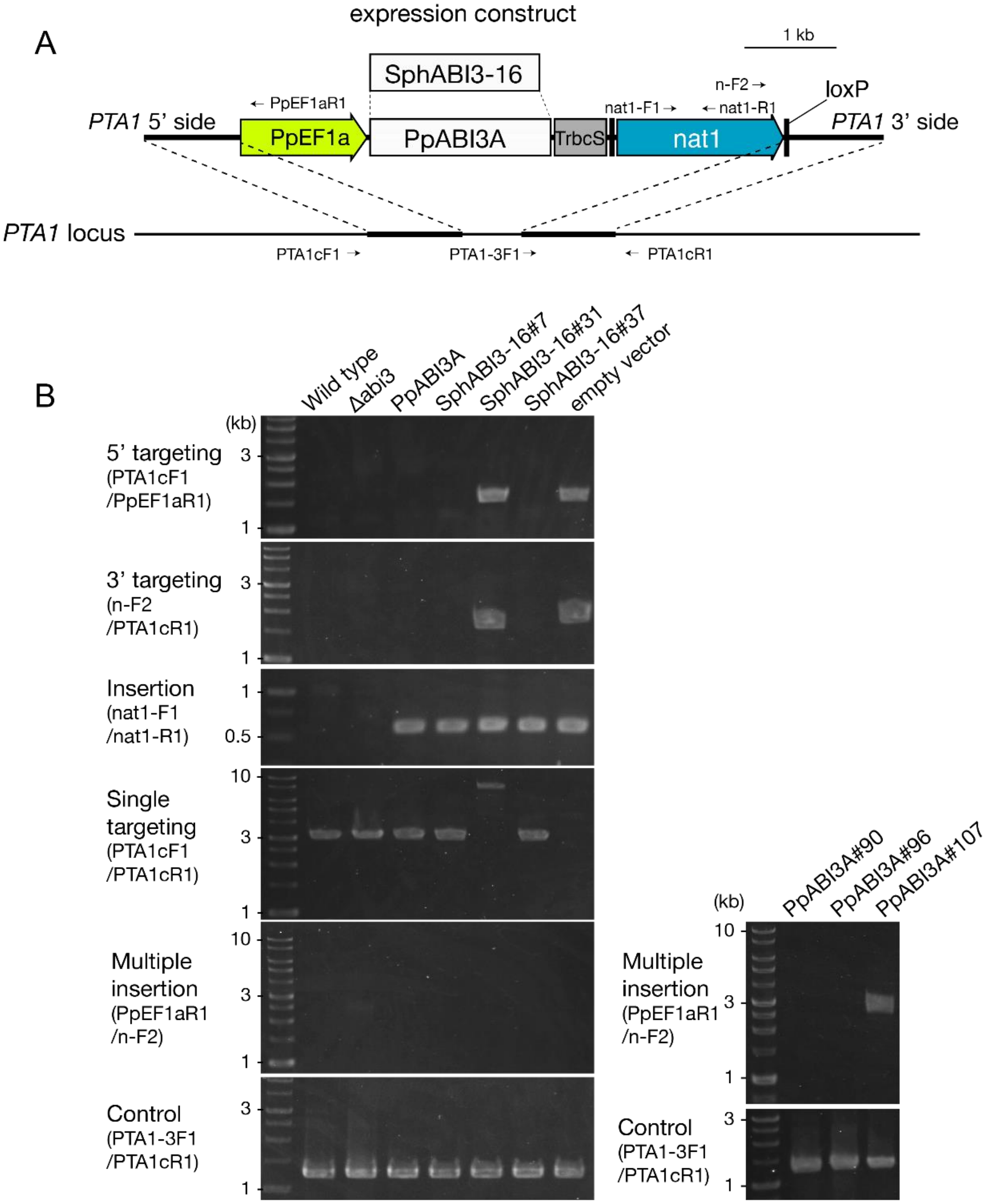

Supplementary Fig. S1. Targeting of the *PpABI3A* and *SphABI3-16* expression constructs. (A) Schematics of the targeting of the expression construct into the *PTA1*-targeting locus. Light green and blue arrows denote a *Physcomitrium patens* EF1a promotor (PpEF1a) and the nourseothricin resistance cassette (nat1), respectively. White, gray, and black boxes show the DNA fragment encoding PpABI3A or SphABI3-16, the pea rbcS3A terminator (TrbcS), and loxP, respectively. Thick bars indicate targeting region of the *PTA1* locus. Small arrows represent the primers (PTA1cF1, PpEF1aR1, PTA1-3F1, nat1-F1, nat1-R1, n-F2, PTA1cR1) for the PCR-based targeting check. (B) PCR to checking the targeting of the inserts. The panels show the PCR-amplified DNA fragments for 5' targeting and 3'targeting to the *PTA1* locus, the presence of the insertion, the detection of the insertion being single or multiple, and the PCR control as indicated. The side panel includes PCR controls for the multiple insertion PCR using a line that shows multiple insertions. PpABI3A#90 is the PpABI3A line used in the main panel.

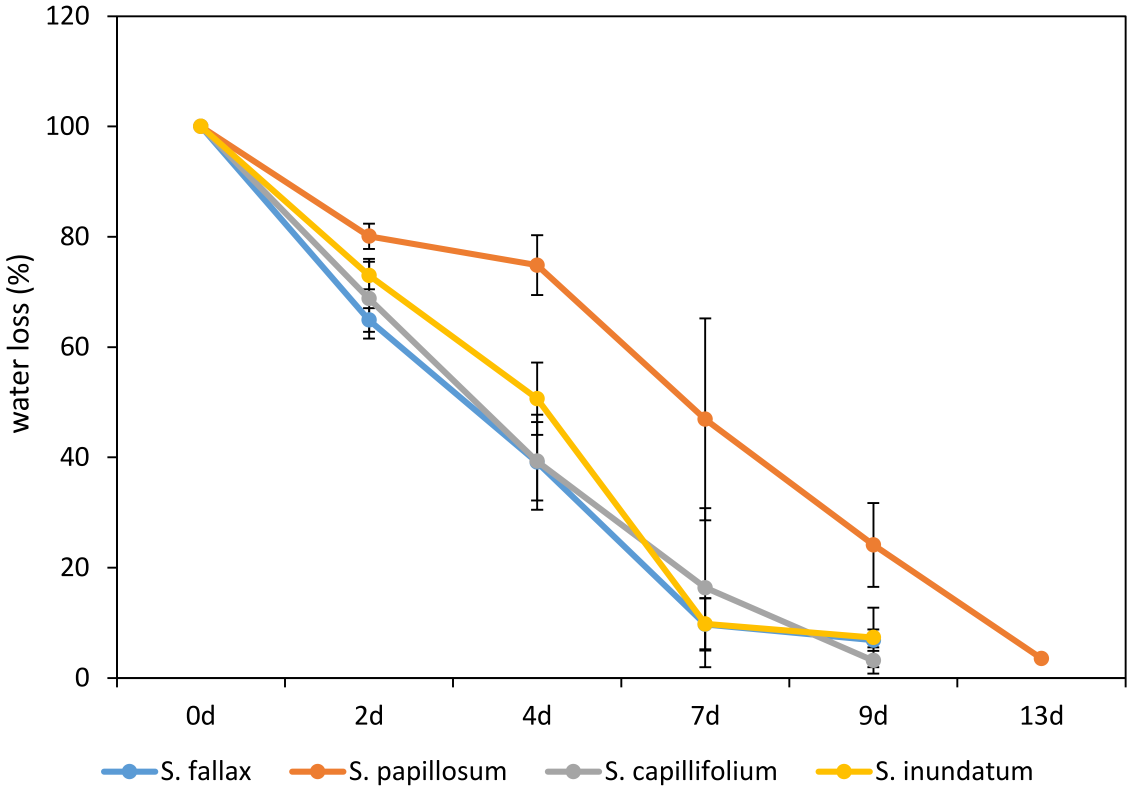

Supplementary Fig. S2. Percentage fresh weight during the desiccation experiment for the four different *Sphagnum* species as indicated in the graph legend. The cosms were weighed on the days indicated in the horizontal axis. Percentage water loss was calculated by dividing the final plant weight by the original plant weight for each time point and multiplying by 100. Data represent averages ± SD of four replica plates.

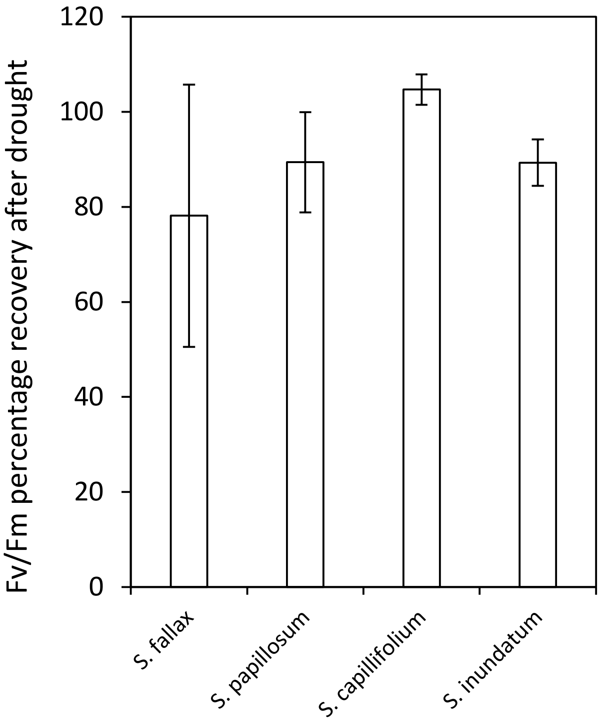

Supplementary Fig. S3. Chlorophyll fluorescence recovery after desiccation as measured by Fv/Fm. Recovery was calculated by dividing the Fv/Fm value after re-watering with the Fv/Fm measured at the start of the drought and expressed as a percentage.

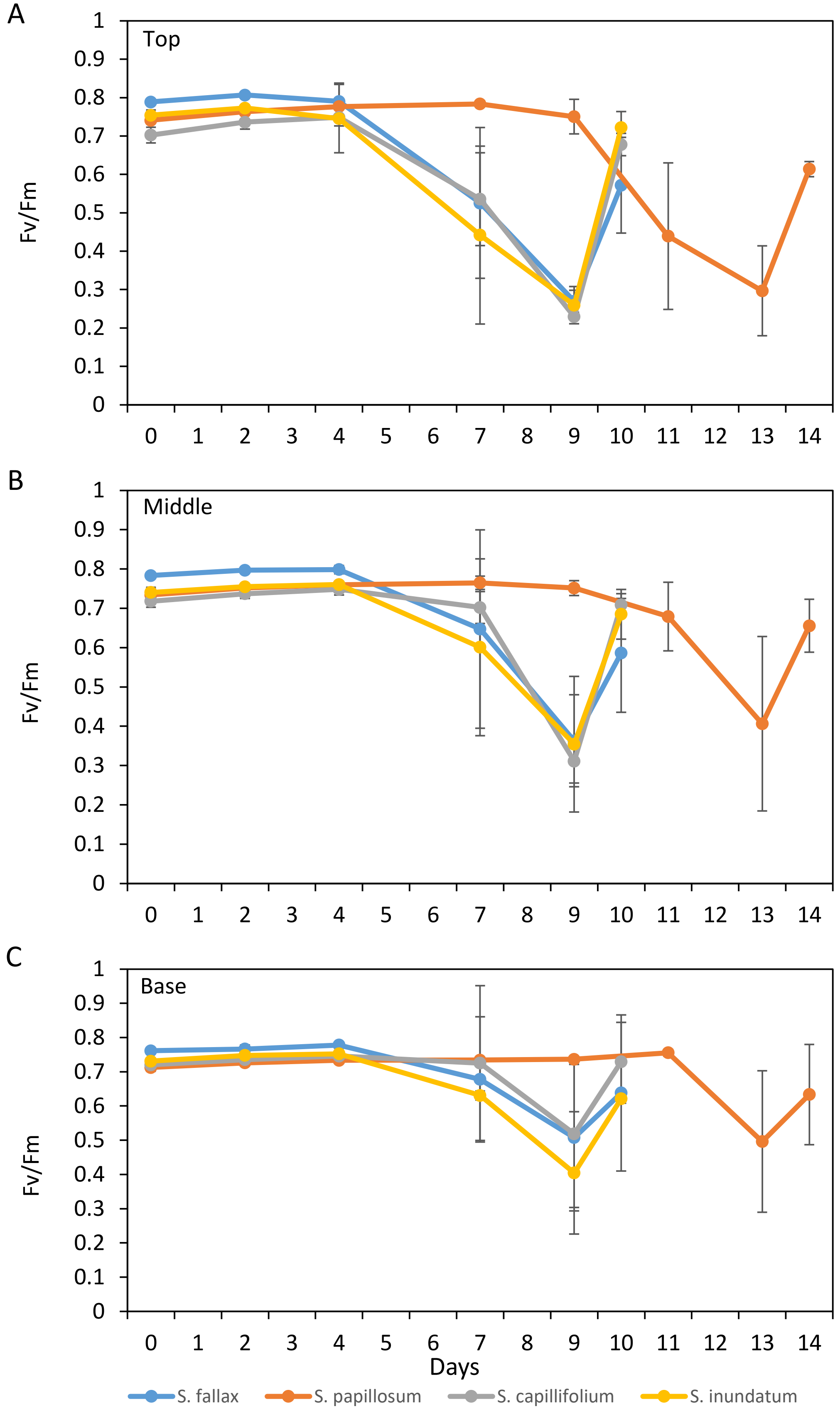

Supplementary Fig. S4. Changes of chlorophyll fluorescence upon desiccation in different regions of the *Sphagnum* plants for the four different species. After imaging, plates were divided into; A top region (containing mostly capitula), B middle region (green, active region) and C bottom (senescing area) and the Fv/Fm value for each region determined. Data represent averages ± SD of four replica plates.

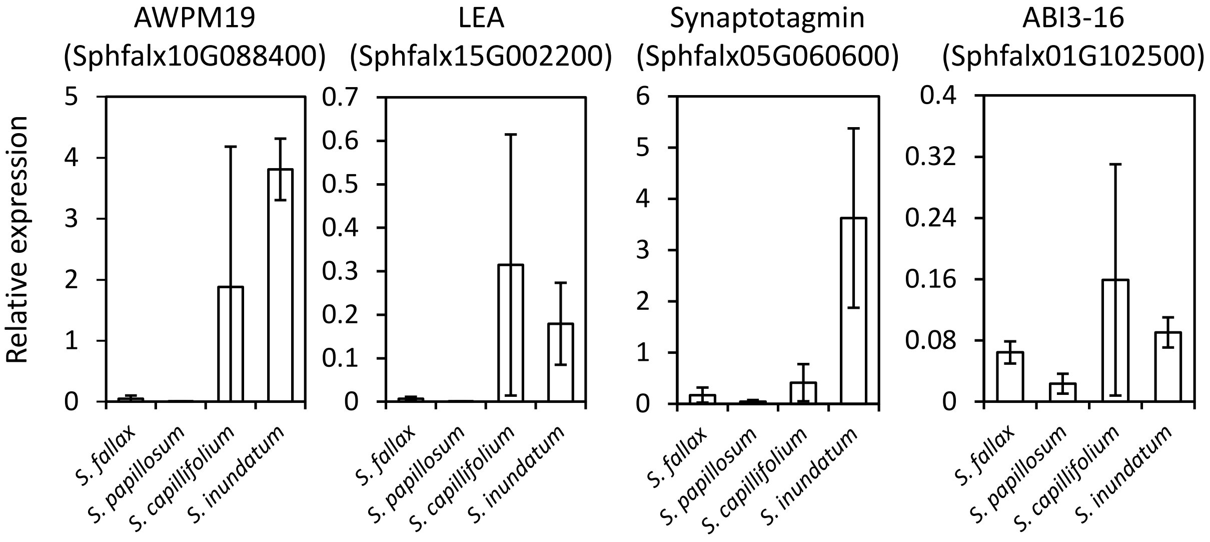

Supplementary Fig. S5. Basal expression of drought responsive genes is higher in *S. inundatum*. Expression of AWPM19 (Sphfalx10G088400), LEA (Sphfalx15G002200), Synaptotagmin (Sphfalx05G060600) and ABI3-16 (Sphfalx01G102500) was determined in non-droughted material by qPCR using EF1α (Sphfalx03G087000) GAPDH (Sphfalx16G076000) as references. Each sample was done in triplicate and data represent average ± SE of three biological replicates.

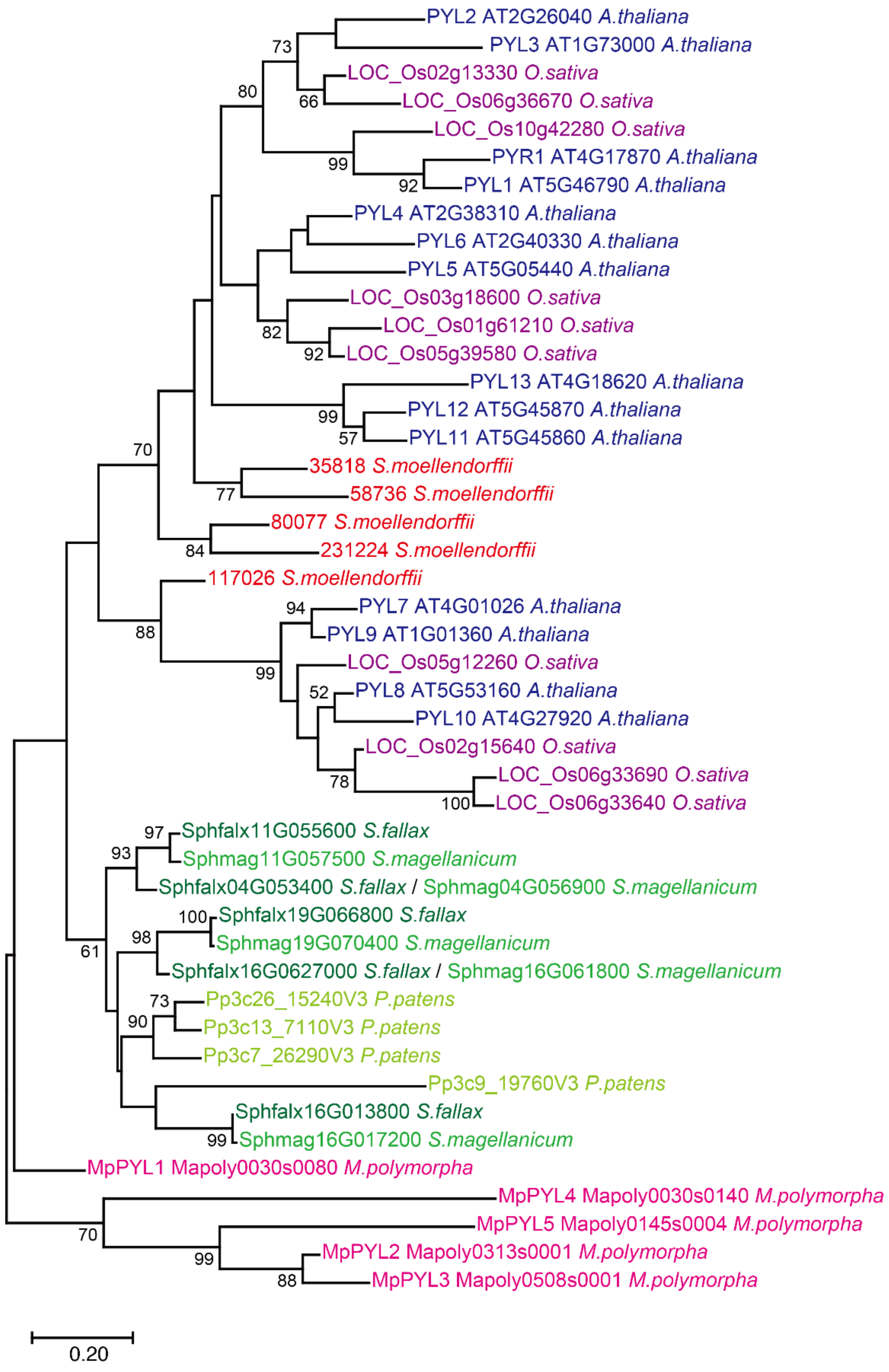

Supplementary Fig. S6. ABA signalling components are present and evolutionarily conserved in *Sphagnum* including the ABA-receptor family PYR1/PYL.

The phylogenetic tree of the PYR1/PYL family was constructed using the Maximum Likelihood method using the JTT matrix. This is an unrooted tree and the tree with the highest log likelihood is shown. Bootstrap values of >50% are shown on the branches. Horizontal branch length is proportional to the estimate evolutionary distance. Genes with identical amino acid sequence within the region used for the phylogenetic analysis are described in parallel.

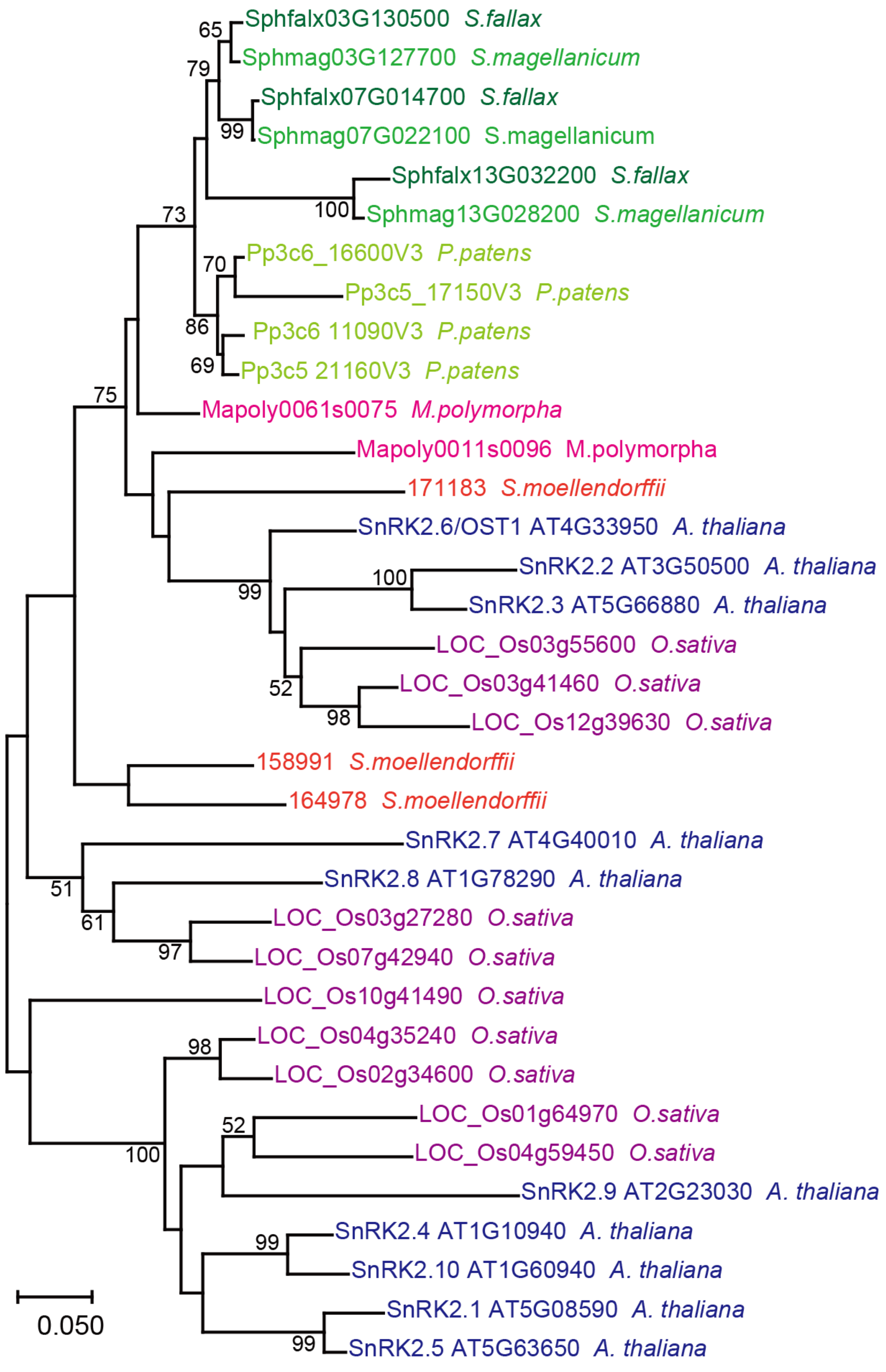

Supplementary Fig. S7. ABA signalling components are present and evolutionarily conserved in *Sphagnum* including SnRK2.
The phylogenetic tree of the SnRK2 family was constructed using the Maximum Likelihood method using the JTT matrix. This is an unrooted tree and the tree with the highest log likelihood is shown. Bootstrap values of >50% are shown on the branches. Horizontal branch length is proportional to the estimate evolutionary distance.

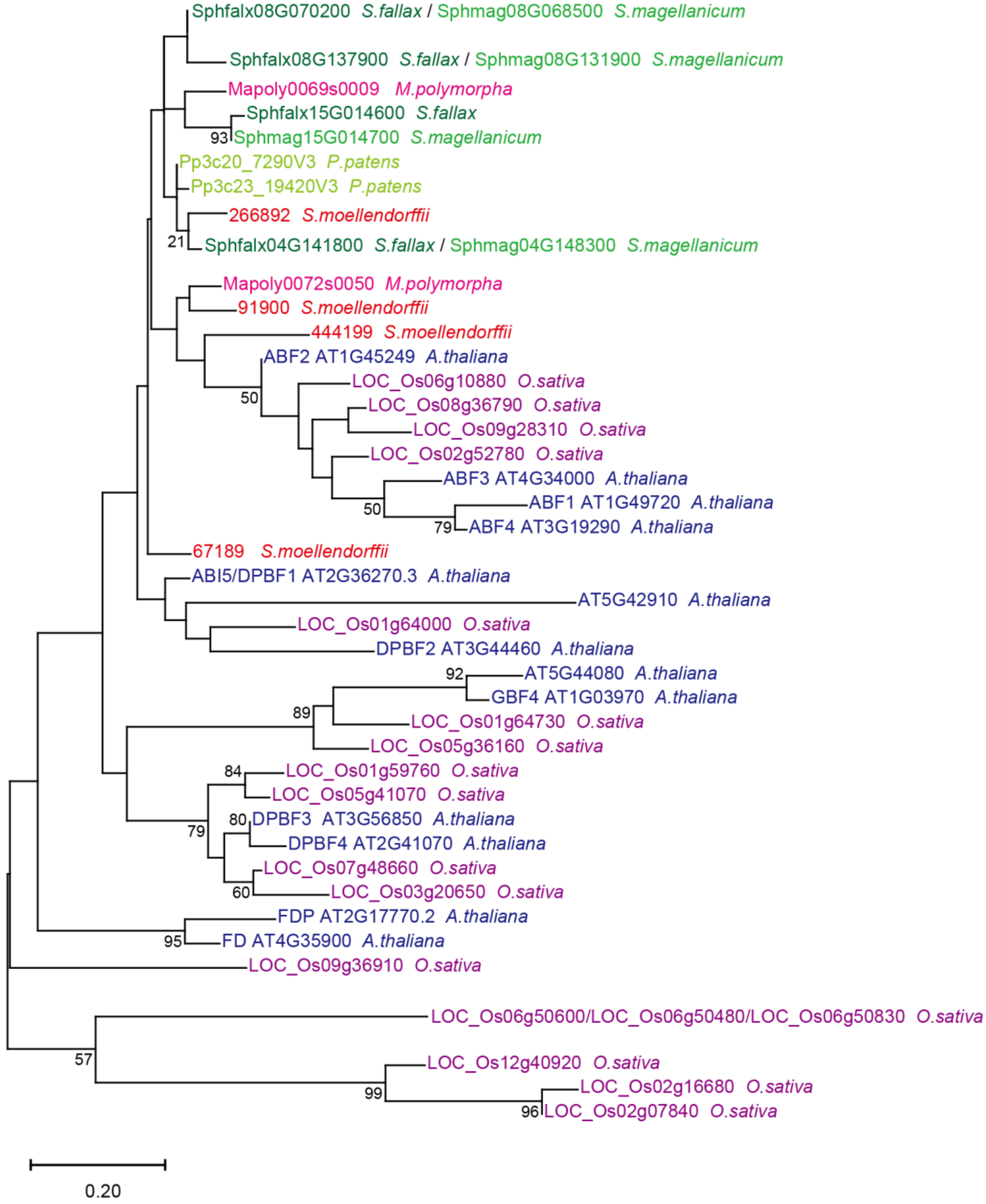

Supplementary Fig. S8. ABA signalling components are present and evolutionarily conserved in *Sphagnum* including ABI5 and ABI5-related proteins.
The phylogenetic tree of the ABI5 family was constructed using the Maximum Likelihood method using the JTT matrix. This is an unrooted tree of group A bZIPs. The tree with the highest log likelihood is shown. Bootstrap values of >50% are shown on the branches. Horizontal branch length is proportional to the estimate evolutionary distance.

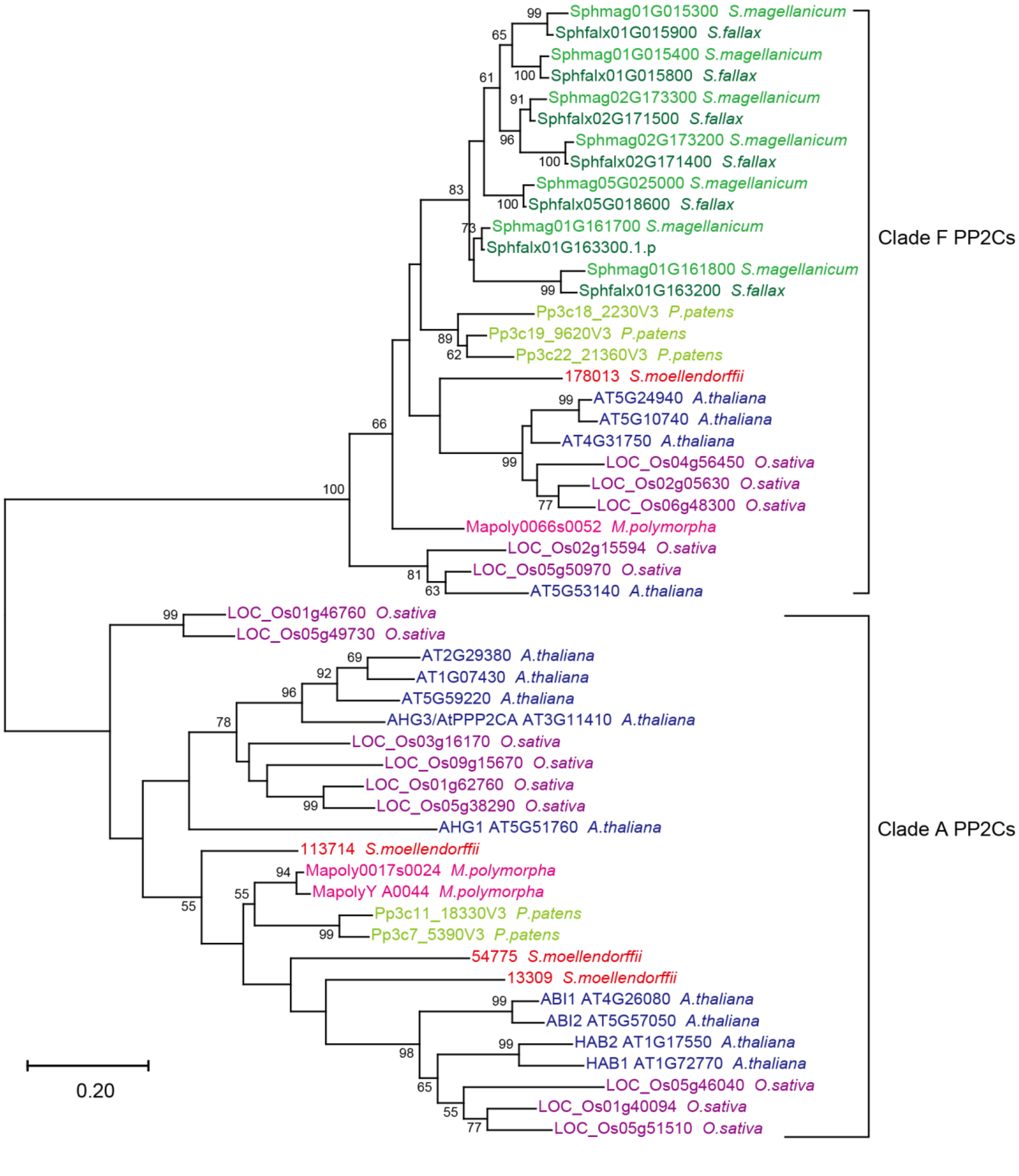

Supplementary Fig. S9. Clade A PPC2A homologues are absent in *Sphagnum*.

The phylogenetic tree was constructed using the Maximum Likelihood method using the JTT matrix. This is an unrooted tree and the tree with the highest log likelihood is shown. Bootstrap values of >50% are shown on the branches. The clade names follow Bhaskara et al., 2019. Horizontal branch length is proportional to the estimate evolutionary distance. Clade F orthologues were detected for all the species tested. Note that clade F of PP2C does not contain full gene members due to the E-value threshold used. We were unable to identify Clade A homologues in *Sphagnum*.

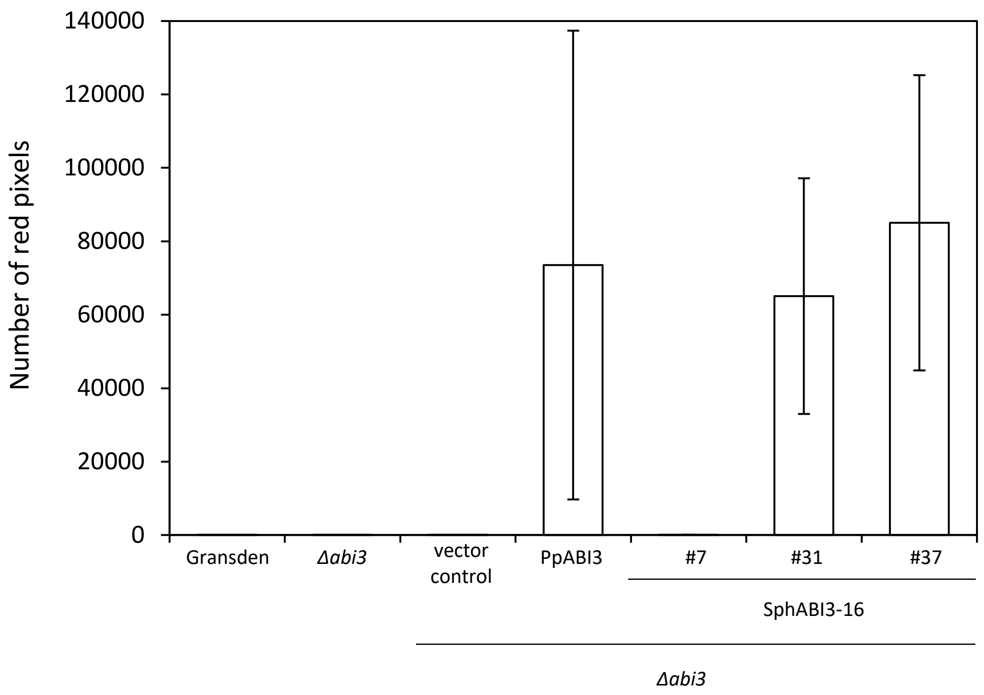

Supplementary Fig. S10. *P. patens* plants with increased expression of *ABI3* show signs of stress as measured by the presence of red pixels. Protonema was grown in cellophane disks overlaid on BCD plates. After seven days, it was transferred to BCD media supplemented with 25µM ABA and imaged after 14days. The number of red pixels in the image was calculated per plate. Data represent averages ± SE of three replica plates.
